# Multiomic profiling of responses to clinical and novel bisphosphonates reveals extraskeletal effects on ageing related signatures

**DOI:** 10.1038/s41392-026-02799-x

**Published:** 2026-07-15

**Authors:** Jinsen Lu, Srinivasa Rao Rao, Helen Knowles, Haoqun Zhan, Beatriz Gamez, Mingyu Qin, Eleanor Platt, Lucy R. Frost, Tiffany-Jayne Allen, Gayle Marshall, Kilian V. M. Huber, Ludwig G. Bauer, Iolanda Vendrell, Darragh P. O’Brien, Benedikt Kessler, Anne Horne, Ian R. Reid, Chas Bountra, James L. Kirkland, Sundeep Khosla, Frank H. Ebetino, Emilio Roldan, R. Graham G. Russell, James R. Edwards

**Affiliations:** 1 Botnar Research Centre, Nuffield Department of Orthopaedics, Rheumatology and Musculoskeletal Sciences, University of Oxford, Oxford, UK; 2 Nuffield Department of Surgical Sciences, University of Oxford, Oxford, UK; 3 Department of Biochemistry, University of Oxford, Oxford, UK; 4 Medicines Discovery Catapult, Cheshire, UK; 5 Target Discovery Institute, Nuffield Department of Medicine, University of Oxford, Oxford, UK; 6 Centre for Medicines Discovery, Nuffield Department of Medicine, University of Oxford, Oxford, UK; 7 Faculty of Medical and Health Sciences, University of Auckland, Auckland, NZ New Zealand; 8 Center for Advanced Gerotherapeutics, Cedars-Sinai Health Sciences University, Los Angeles, USA; 9 Robert and Arlene Kogod Center on Aging, Mayo Clinic, Rochester, MN USA; 10 BioVinc LLC, Pasadena, CA US; 11 Department of Chemistry, University of Rochester, Rochester, NY USA; 12 Qualix DoT, Barcelona, Spain; 13 Mellanby Centre for Bone Research, Division of Clinical Medicine, School of Medicine and Population Health, University of Sheffield Medical School, Sheffield, UK

**Keywords:** Drug screening, Senescence, Preclinical research

## Abstract

Bisphosphonates (BPs) have been used effectively to treat excessive bone loss for over 50 years. Recent clinical evidence suggests extra-skeletal benefits but how this occurs remains unknown. Here we use a panel of human, murine and cellular assessments to chart BP-induced ageing-related changes both systemically and at local organ sites. In vivo spatial transcriptomics in aged mice treated with zoledronate showed a shift in cellular composition towards that of young animals specifically in heart, liver and intestine, with upregulation of genes governing detoxification, mitochondrial stability, energy metabolism, and antioxidation. A 5000-plex randomized trial based human proteomic analysis showed significant alterations in ~400 proteins after zoledronate treatment, with downregulation of proteins linked to genomic instability, proteostasis loss, mitochondrial dysfunction, stem cell exhaustion, and SASPs. Fluorescent labeling and tracing confirmed uptake of bisphosphonates by non-skeletal cells. In addition, low doses of several common, clinically utilized BPs stimulated growth and protected against DNA damage-induced senescence in multiple human cell types, with strongest effects in cardiomyocytes. Finally, proteome-wide target deconvolution with AlphaFold identified previously unrecognized binding partners, including PHB2 and ASAH1, and downstream upregulation of MEF2A was validated to be a key mediator of zoledronate triggered benefits in cardiomyocytes. Collectively, these results identify potential geroprotective mechanisms for BP action in multiple non-skeletal tissues.

## Introduction

Diverse ageing-related pathologies may share a common molecular etiology, driven by fundamental hallmarks such as cellular senescence, genomic instability, impaired proteostasis and mitochondrial dysfunction. Consequently, a single therapeutic intervention targeting these core mechanisms may provide broad-spectrum protection against multiple morbidities simultaneously (the Geroscience hypothesis).^1,2^ The bisphosphonate (BP) class of drugs has been used worldwide as an effective treatment for disorders of excessive bone loss (e.g., osteoporosis, cancer-induced osteolysis) over the past 50 years.^3^ Chemically, BPs are stable analogues of pyrophosphate in which the P-O-P bond is replaced by a non-hydrolysable P-C-P backbone. This seemingly simple structural change confers resistance to enzymatic degradation and a unique ability to bind calcified surfaces which, coupled with their ability to inhibit bone resorption, has led to several different BPs being developed for clinical use.^3^ The earliest BPs to be used were clodronate (CLO) and etidronate (ETI), which act through different biochemical mechanisms to more recently developed nitrogen-containing BPs (N-BPs) such as pamidronate (PAM), alendronate (ALN), risedronate (RIS), ibandronate (IBN), and notably zoledronate (ZOL).^4^ The bone-resorbing osteoclast is the primary bone cell capable of degrading and removing large quantities of bone.^5^ Non-nitrogen containing BPs are metabolised intracellularly into cytotoxic ATP analogues that disrupt osteoclast energy metabolism and promote apoptosis. In contrast, incorporation of nitrogen into the BP structure confers substantially greater anti-resorptive potency and, in some agents, stronger bone mineral affinity, resulting in improved clinical efficacy.^6,7^ The ingestion of large quantities of bone-bound N-BP triggers an inhibition of farnesyl pyrophosphate synthase (FPPS), a key enzyme in the mevalonate pathway governing cholesterol biosynthesis impairing the prenylation of multiple GTPases to alter intracellular signalling events, and induce osteoclast apoptosis.^8,9^ In addition to clinically established BPs, other analogues such as lidadronate (IG9402) can also function through mechanisms distinct from those of classical BPs. In particular, IG9402 appears to preserve osteoblast and osteocyte viability through connexin 43-dependent signalling and activation of Src/ERK pathways, thereby promoting bone cell survival and potentially improving bone quality.^10^

More recently, studies of patients receiving BPs for skeletal-related conditions have revealed a reduced mortality^4,11–14^ and a reduction in the development of a number of disorders commonly associated with ageing, such as cardiovascular and respiratory disorders, cancer, along with improved overall survival following admission to intensive care units.^4,8,9,11^ Such beneficial effects cannot be accounted for by the prevention of bone loss alone, challenging the bone- and osteoclast- specific nature of BP activity. Preclinical studies further support the pleiotropic potential of BP action beyond the skeleton. Reported non-bone effects include modulation of monocyte and macrophage biology, inhibition of angiogenesis and tumour cell invasion, and activation of γδ T-cell responses through accumulation of phospho-antigens downstream of mevalonate pathway blockade.^15–18^ Importantly, the longstanding view that BPs act only at mineralised surfaces is now being expanded. Cellular uptake has been documented in osteoclast lineage cells, monocytes, macrophages, endothelial cells, kidney cells and multiple tumour cells. For N-BPs, delivery to the cytosolic target pool appears to involve fluid-phase endocytosis followed by lysosomal export mediated by the SLC37A3-ATRAID transporter complex.^19^ These observations raise the possibility that BPs may impact other cell types beyond osteoclasts, which may influence processes relevant to ageing, including vesicular trafficking, inflammatory signalling, oxidative stress responses and autophagy, The mechanisms underlying these effects remain largely unknown, indicating that the pleiotropic pharmacology of BP action remains incompletely understood and potential polypharmacology of BPs untested.

Cellular senescence is a central component of the ageing process and encompasses several major forms, including replicative senescence, oncogene-induced senescence, therapy-induced senescence, and stress-induced premature senescence.^20^ Despite this diversity, persistent DNA damage and sustained DNA-damage response signalling constitute a common and fundamental trigger mechanism across multiple senescence programmes. This persistent stress response ultimately enforces stable cell-cycle arrest through p53/p21 and p16INK4a/RB signalling, while allowing cells to remain metabolically active.^21^ These cells often develop a senescence-associated secretory phenotype (SASP), comprising pro-inflammatory cytokines, chemokines, growth factors and matrix-remodelling proteases that propagate paracrine inflammation and tissue dysfunction.^22^ Early preclinical studies suggest BPs may display both senomorphic and context-dependent senolytic-like activities. Nevertheless, the relevant BP doses, target cell populations, molecular mediators and direct binding partners beyond FPPS remain incompletely defined.^23,24^

With the development of multi-omics technologies such as aptamer-based plasma proteomics, two-dimensional thermal proteome profiling, and spatial transcriptomic profiling, it is currently possible to interrogate systemic protein changes, unbiased drug-target interactions, and tissue-contextual molecular responses at high throughput.^25–27^ The complex biology of BP action can therefore be captured more effectively by integrating multimodal data across human, animal and cell-based experiments. This study explores the non-skeletal effects of BPs and their potential role on ageing signatures in vivo and in vitro, using an integrated multiomics approach, including protein-binding, as well as alterations to the human secretome, transcriptional control and cellular impact.

## Results

### Bisphosphonate treatment is associated with reduced disease progression and severity

**Fig. 1:**
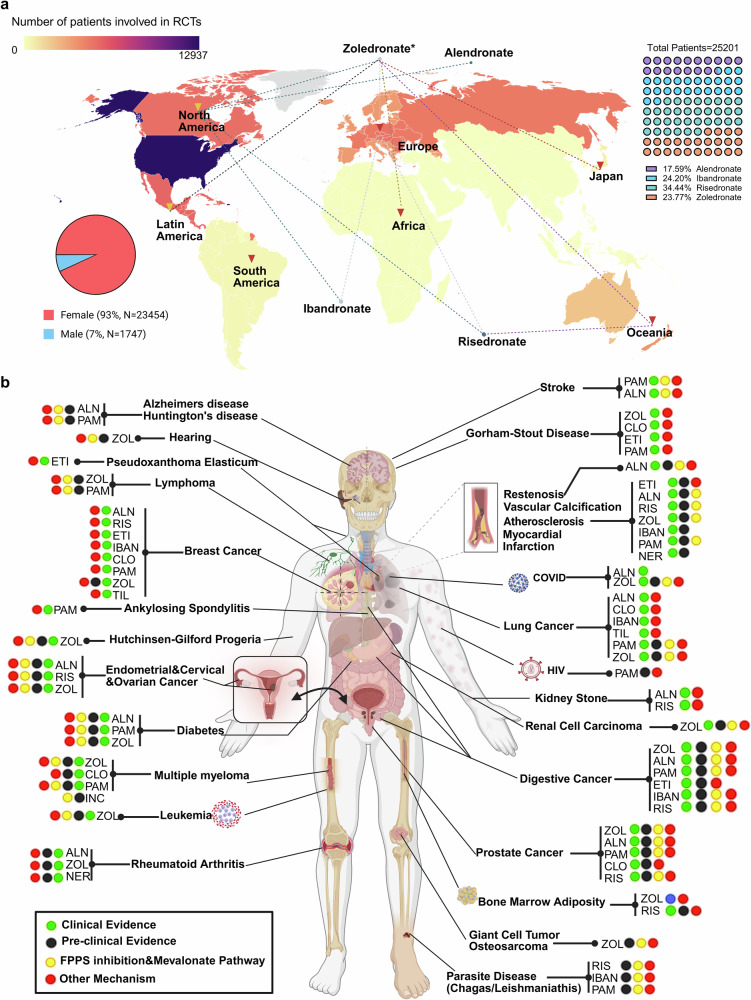
Clinical and preclinical evidence associated with bisphosphonates related medical benefits. **a** Geographical heatmap depicting global distribution of RCTs related to mortality reduction conferred by BPs. Details of RCTs in Suppl. Table 1. Heatmap = number of patients in a specific region. Colour = BP. RCT regions labelled and linked by dotted line. Gender distribution of participants patients illustrated as pie chart. **b** Body based illustration of BP benefit against disease. Details of evidence in Suppl. Table 2. Green = clinical study, Black=pre-clinical study, Yellow = benefit attributed to FPPS/mevalonate pathway inhibition, Red = other mechanism. PAM Pamidronate, IBA Ibandronate, TIL Tiludronate, NER Neridronate, INC, Incadronate. *p < 0.05 vs placebo group (mortality rate reduction)

A series of bone loss-related randomized controlled trials^12–14,28–39^ across the globe suggest BP treatment was related to less lethal events beyond bone, with RIS the most frequently tested (34.44%), and ZOL (23.77%) showing the greatest significant benefit, along with IBN (24.2%) and ALN (17.59%), in primarily female cohorts across all continents (Fig. 1a, Suppl. Table 1). Furthermore, a descriptive summary of heterogenous BP treatment ‘studies (clinical and pre-clinical)^29–95^ revealed associations with improved outcomes for diseases at diverse organ and tissue sites (Fig. 1b, Suppl. Table 2). This included cardiovascular disease (e.g. vascular calcification, stroke, atherosclerosis, myocardial infarction), cancers (e.g. oesophageal, colorectal, prostate, breast, multiple myeloma, osteosarcoma, cervical, and ovarian), diabetes, neurodegenerative disorders, hearing loss, hereditary diseases such as Pseudoxanthoma Elasticum (PXE), Hutchinson-Gilford progeria syndrome, parasitic infections (Chagas disease, leishmaniasis), and rheumatic diseases (ankylosing spondylitis, rheumatoid arthritis).

### Zoledronate treatment alters plasma proteome in female osteopenic patients

**Fig. 2:**
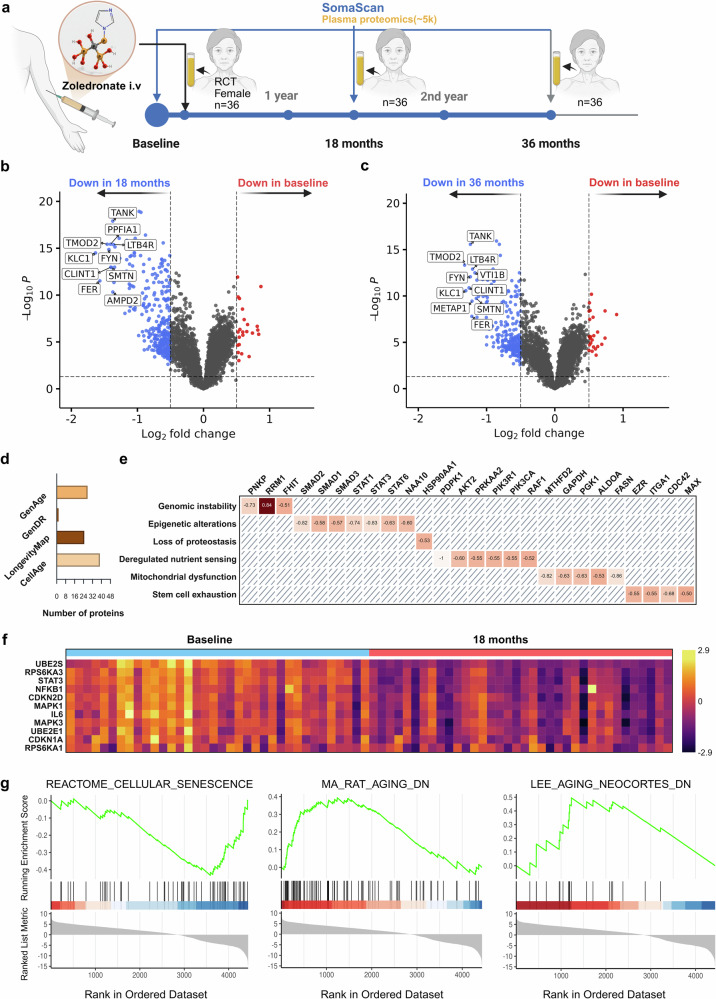
Plasma proteomic changes in osteopenic women treated with zoledronate. **a** Schematic overview of the study design. Plasma samples from 36 osteopenic female patients, selected from a larger randomized controlled trial, were collected at baseline and after 18 and 36 months of zoledronate treatment. Proteomic profiling was performed using the SomaScan platform (~5000 proteins detected per sample). **b**, **c** Volcano plots showing differential plasma protein expression after 18 and 36 months of treatment. Detailed protein list in supplementary Data. 1. **d** Mapping of altered plasma proteins (APPs) to the Human Ageing Genomic Resources (HAGR) database at 18 months. Detailed results in Suppl. Table 3. **e** Heatmap of top APPs associated with anti-ageing processes. **f** Heatmap of senescence-associated secretory phenotype (SASP) proteins identified among APPs at 18 months. **g** Enrichment score (ES) plots of top gene set enrichment analysis (GSEA) pathways related to ageing and senescence

Plasma samples collected from female osteopenic patients^96^ before and after receiving a ZOL infusion (5 mg, 18 and 36 months), were assessed for changes in systemic protein levels (SomaScan, 5000-plex, SomaLogic) (Fig. 2a). A significant number of proteins showed altered circulating levels 18 and 36 months following treatment with ZOL (Fig. 2b, c). The highest regulated individual proteins included KLC1, FER, TMOD2, FYN, PPFIA1, CLINT1, SMTN, TANK, AMPD2, and LTB4R (supplementary Data. 1). A substantial fraction of altered circulating proteins correlated with ageing-related signatures in the Human Ageing Genomic Resources (HAGR) database (Fig.2d and Suppl. Table 3) including downregulated markers of genomic instability, proteostasis loss, deregulated nutrient sensing, mitochondrial dysfunction and stem cell exhaustion (Fig. 2e). Furthermore, several downregulated proteins were identified as common members of the toxic cocktail released by senescent cells known as the senescence-associated secretory phenotype (SASP) (including inflammatory mediators IL-6, NFĸB and STAT3, senescence enforcer CDKN1A and CDKN2D), suggesting a seno-modifying potential for ZOL in mid-/older-aged females (Fig. 2f). In addition, GSEA revealed that proteins downregulated by ZOL were significantly enriched in gene sets related to cellular senescence and brain ageing (Fig. 2g).

### Zoledronate induces localised transcriptomic changes in ageing-related pathways in vivo

**Fig. 3:**
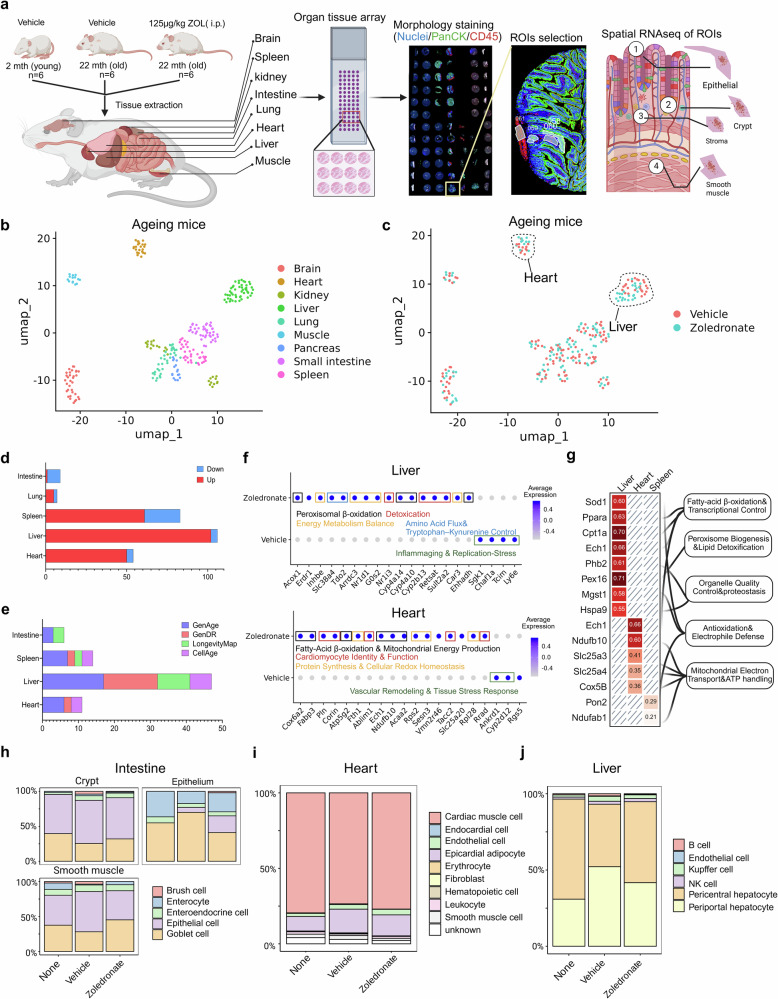
Organ spatial transcriptomic profiling of zoledronate effects in ageing mice. **a** Schematic overview of animal study design. Three groups of mice (*N* = 6 per group), including one young control group and two aged groups, were treated with vehicle or zoledronate for 2 months. Eight representative organs were collected, processed into tissue arrays, and subjected to morphological staining and region-of-interest (ROI) selection for spatial RNA sequencing (Suppl. Table 4). **b** UMAP clustering of transcriptomic profiles across organs in aged mice. **c** UMAP plots comparing transcriptomic profiles of organs in aged mice treated with vehicle versus zoledronate. **d** Bar plot of differentially expressed genes (DEGs) across organs between vehicle- and zoledronate-treated aged mice (detailed in Suppl. Tables 5, 6). **e** Bar plot of DEGs mapped to the Human Ageing Genomic Resources (HAGR) database across organs. **f** Top DEGs identified in liver and heart between treatment groups. **g** Representative ageing-beneficial DEGs in liver, heart, and spleen. **h**–**j** Cell type deconvolution analyses showing shifts in cell fractions in intestine, heart, and liver

The significant shift in the plasma proteome reflects a systemic ZOL-induced alteration within the blood compartment of female osteopenic patients but on its own, does not provide insight into BP effects upon individual organs or the potential for BP-induced transcriptomic changes. To test the transcriptional impact of ZOL outside of the skeleton, 8 non-skeletal tissues were collected from aged (24mth, F) mice following ZOL treatment (2 months, 125 μg/kg or vehicle control) and compared for local alterations in gene signatures. In each individual tissue, representative ROI across organs were selected using IF-stained (CD45, panCK) sections based on key tissue-specific regions followed by RNAseq via the GeoMx platform (Fig. 3a (e.g., intestine) and Suppl. Table 4). UMAP analysis revealed that ROI samples from aged mice displayed distinct organ-specific transcriptomic profiles (Fig. 3b). Zoledronate treatment produced significant profile shifts in liver and heart tissues compared with vehicle controls (Fig. 3c), consistent with the differential gene expression patterns observed across organs (Fig. 3d). Similarly, the highest tissue specificity of significantly altered plasma proteins in female patients was observed in the stomach, cardiac and skeletal muscle, bone marrow, cerebral cortex, liver, and testis, which is consistent with murine data (Suppl. Fig. 1). In the liver, the top differentially expressed genes (DEGs) after ZOL treatment were predominantly upregulated and involved in peroxisomal β-oxidation, detoxification, energy balance, amino acid flux, and tryptophan metabolism, whereas downregulated genes were enriched for pathways associated with inflammaging and replication stress (Fig. 3f and Suppl. Table 5). In the heart, ZOL induced upregulation of genes controlling fatty acid β-oxidation, mitochondrial energy production, cardiomyocyte function, protein synthesis, and redox homeostasis, while downregulated genes were linked to vascular remodeling and stress responses (Fig. 3f and Suppl. Table 6). In the intestine, ZOL treatment significantly downregulated lipid and cholesterol metabolism pathways, suggesting a potential suppression of age-associated hyperlipidemia or metabolic drift. In the lung, DEGs exhibited an enrichment of lipoprotein transport and steroid biosynthesis (Suppl. Fig. 2). Across liver, heart, spleen, and intestine, 53 DEGs mapped to the HAGR database (Fig. 3e), including 15 upregulated genes associated with beneficial processes such as fatty acid β-oxidation, peroxisome biogenesis, lipid detoxification, organelle quality control, proteostasis, mitochondrial electron transport, and antioxidation (Fig. 3g). Interestingly, the cell deconvolution results indicate that the organ-based cell landscape vastly differed between young and aged mice. However, upon ZOL administration, aged mice exhibited a cell distribution shift towards that of younger animals in heart, liver, and intestine (Figs. 3h and i).

### Bisphosphonate uptake and distribution in non-skeletal cells

**Fig. 4:**
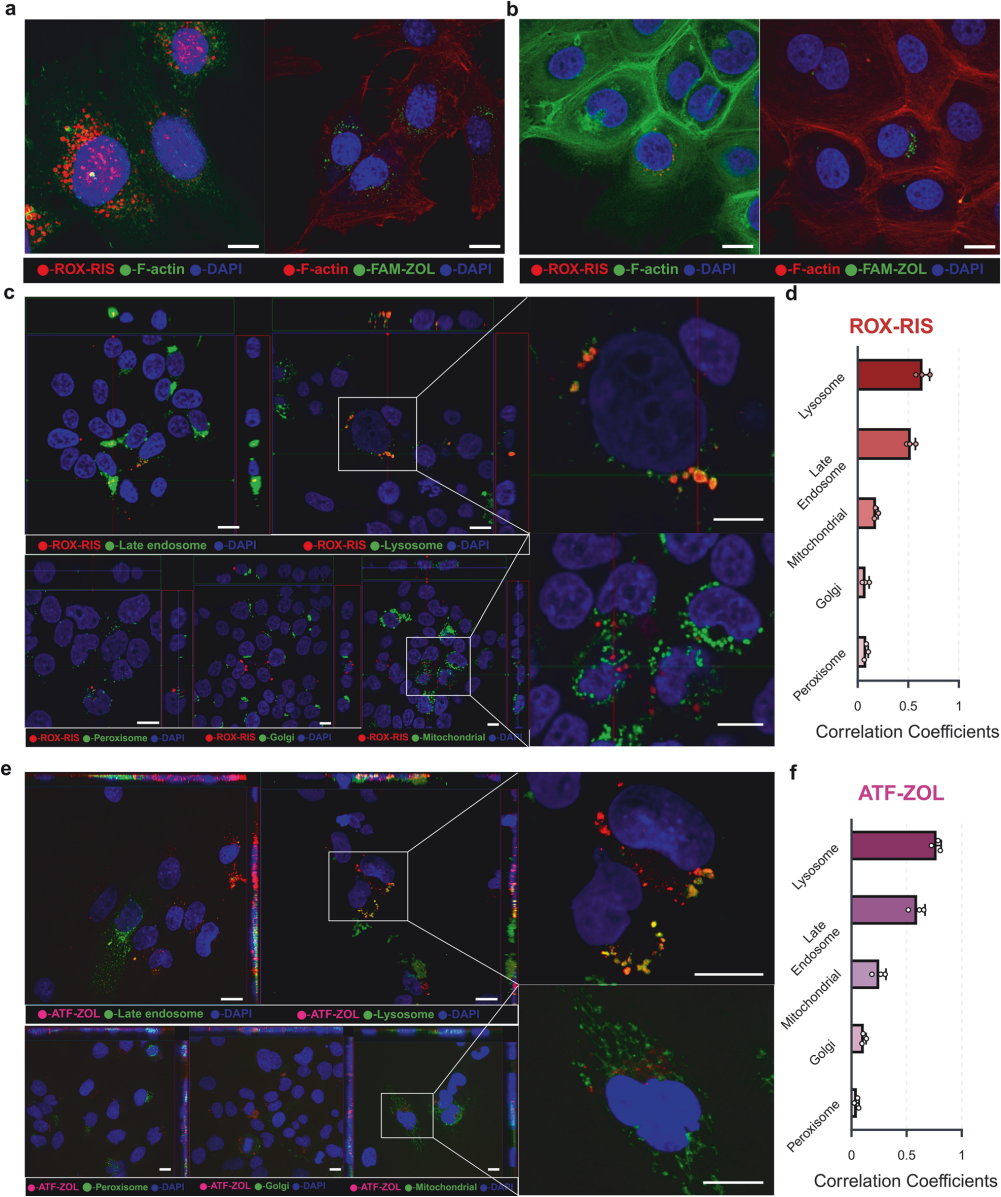
Uptake and subcellular localization of bisphosphonates in non-skeletal cells. **a**, **b** Subcellular distribution of fluorescently labeled bisphosphonates (ROX-RIS and FAM-ZOL) in HUH-7 (**a**) and AC-16 (**b**) cells. **c**–**f** Co-localization of ROX-RIS (**c**) and ATF-ZOL (**e**) with subcellular organelles, including late endosomes, lysosomes, peroxisomes, the Golgi apparatus, and mitochondria, in HEK and AC-1 cells. Corresponding Pearson’s Correlation Coefficients were calculated to assess the overlap between ROX-RIS (**d**) or ATF-ZOL (**f**) and various organelle markers (green) in HEK and AC-16 cells respectively. Scale bar=10μm. Data represent the mean ± SD from three independent replicates of regions showing both drug and organelle signals

Through their binding of calcified surfaces (bone) and internalization by bone-resorbing osteoclasts, the effects of BPs have been thought to be primarily restricted to these cells alone. However, other phagocytic cell populations of the bone microenvironment (and beyond) may also have potential for BP internalization^97,98^ with possible effects outside of the skeleton.^99^ Having demonstrated systemic proteomic and local transcriptomic effects following BP treatment in situ, we sought to validate and visualize the internalization of BPs within a panel of non-skeletal human cell types. Fluorescently labeled RIS (ROX-RIS) and ZOL (FAM-ZOL, ATF-ZOL) were used alongside viral based fluorescent organelle markers. Whilst uptake efficiency varied across cell types, both RIS and ZOL were observed within the majority of cells tested (Fig. 4a, b and Suppl. Fig. 4) (0.1 µM), with heart, kidney, and liver cells demonstrating the highest BP content, and indicating that the intracellular availability of BPs might vary among diverse tissues. Co-IF imaging with a panel of intracellular organelle markers revealed a preferential localization of RIS and ZOL with lysosomes and endosomes, suggesting a potential role for BPs in the management of intracellular protein quality, quantity, and modification (Fig. 4c–f). These observations not only underscore a broader cellular accessibility of BPs than previously reported but also hint at potential intracellular targets and pathways that might be influenced.

### Bisphosphonates induce contrasting dose-dependent effects on cell growth

**Fig. 5:**
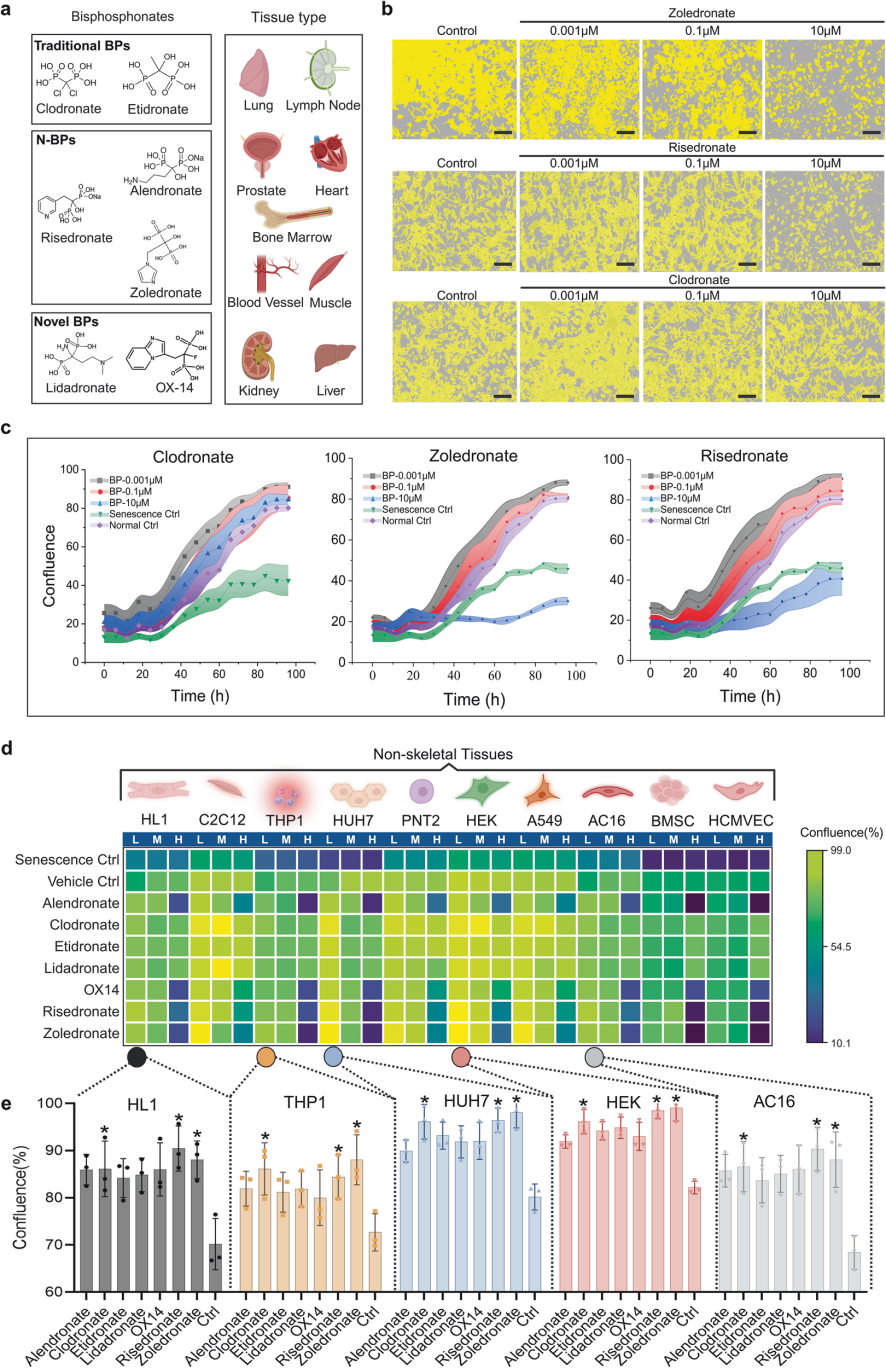
Dose-dependent effects of bisphosphonates on non-skeletal cell growth. **a** Screening workflow for bisphosphonate treatments across a panel of non-skeletal cell types. **b** Representative endpoint confluence images of HUH-7 cells after treatment with Clodronate, Risedronate, and Zoledronate at low (0.001 μM), medium (0.1 μM), and high (10 μM) concentrations for 96 h. Scale bar = 50 μm. **c** Time-course effects of Zoledronate, Clodronate, and Risedronate on HUH-7 cell growth. **d** Heatmap summary of endpoint confluence ratios across the non-skeletal cell panel following treatment with seven representative bisphosphonates at all dosages. **e** Bar plots of endpoint confluence ratios at low-dose treatment of all seven bisphosphonates in AC-16, HL-1, HEK, HUH-7, and THP-1 cells

The internalization of relatively high doses of BPs by osteoclasts during bone resorption (approximately 10 μM) leads to apoptosis.^100^ A panel of human cells were treated with clinical and novel BPs across multiple doses and time points to explore the full cellular impact of BPs. In considering the functional diversity within the BP family, seven typical compounds were selected representing non-nitrogen (non-N) containing BPs (CLO, ETI), nitrogen (N) containing BPs (ZOL, RIS, ALN), and two non-clinically used nitrogen containing BPs (OX14, IG9402 or Lidadronate) with varying bone affinity and effects on FPPS. Compounds were tested in cells across representative tissue types, including those linked to beneficial clinical outcomes (lung, heart, blood vessel, kidney, muscle, liver, prostate, lymph node, and bone marrow) (Fig. 5a). Using live cell imaging (IncuCyte) combined with AI-aided confluence analysis, BP treatment (up to 4 days) at low (0.001 μM), mid (0.1 μM), and high (10 μM) doses revealed contrasting effects across multiple cell types (Fig. 5b, c). At high BP doses, a significant reduction in growth was induced by the majority of BPs tested, similar to that seen with apoptotic control and in agreement with our understanding of BP action in osteoclasts. However, low BP doses (<0.01 μM) were shown to stimulate cell growth. Specifically, low dose treatment with ZOL significantly stimulated proliferation in cardiomyocyte (28%), cardiovascular endothelia (9.4%), renal epithelia (20.5%), hepatocytes (22%), myoblasts (8.7%), and monocytes (21.1%). A similar significant increase was seen with RIS and the non-N BP CLO (Fig. 5d, e). These data suggest that BPs might act upon a variety of cell types, with the potential to confer beneficial effects at lower doses. Importantly, as N- and non-N-containing BPs function through distinct cellular mechanisms, the similar increase in cell growth stimulated by most BPs suggests combined or novel mechanisms might mediate this response.

### Bisphosphonates protect against induction of DNA damage-related senescence

**Fig. 6:**
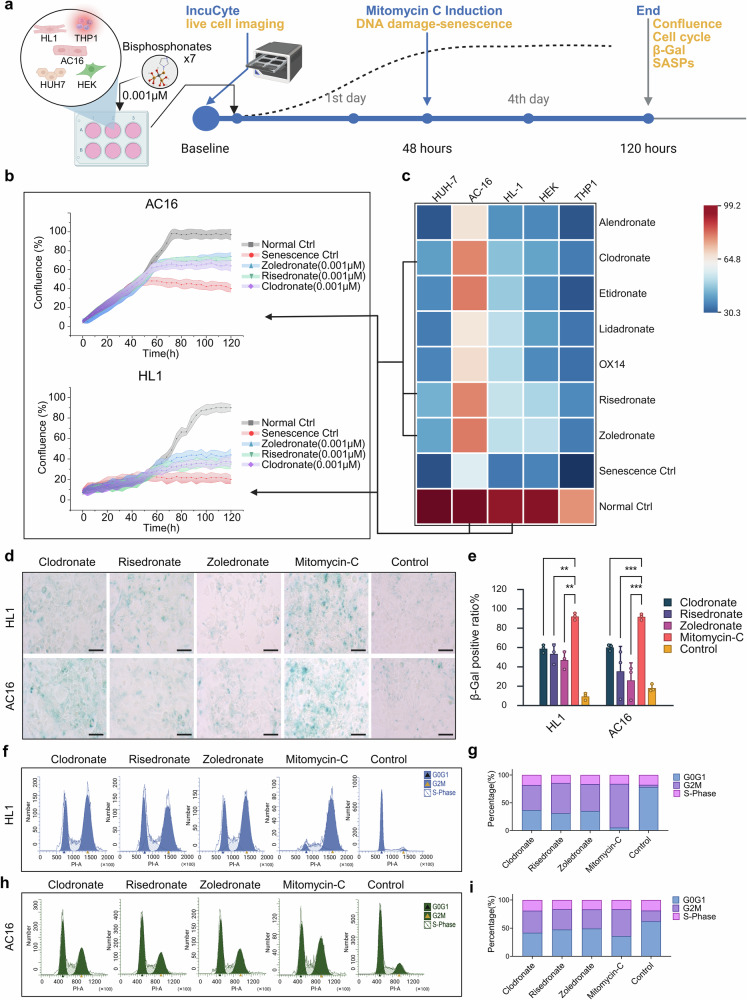
Low-dose bisphosphonates protect non-skeletal cells from DNA damage–induced senescence. **a** Screening workflow to assess the effects of bisphosphonates on mitomycin C–induced senescence in selected non-skeletal cell types. HL-1, THP-1, AC-16, HUH-7, and HEK cells were pretreated with 0.001 μM bisphosphonates for 48 h, followed by mitomycin C exposure to induce senescence. Live-cell imaging was performed with the IncuCyte system for up to 120 h, and senescence was assessed by cell confluence, cell cycle, β-galactosidase staining, and SASP detection. **b** Time-course effects of zoledronate, clodronate, and risedronate pretreatment on AC-16 and HL-1 cell growth following mitomycin C treatment. **c** Heatmap summary of endpoint confluence ratios across all tested non-skeletal cell types after pretreatment with seven bisphosphonates at low dose compared with control. **d**, **e** Representative SA-β-galactosidase staining (**d**, ×200) and quantification (**e**) of AC-16 and HL-1 cells after bisphosphonate pretreatment and senescence induction. **f**–**i** Cell cycle analysis (propidium iodide labelling) of AC-16 and HL-1 cells (**f**, **h**) and corresponding quantification (**g**, **i**) after bisphosphonate pretreatment and senescence induction. *p* < 0.05 versus normal control (NC). Data are presented as mean ± SD, *n* = 3. Scale bar = 50 μm

Expanding upon our observations on ageing and senescence from BP-treated human and murine studies, we tested whether BP pre-treatment might prevent senescence induction in our panel of non-skeletal cells (Fig. 6a). Exposure of HUH-7, AC16, HL1, HEK, and THP1 cells to low dose BP (0.001 μM) prior to induction of DNA damage-triggered senescence, protected against a reduction in confluence, with CLO, RIS and ZOL demonstrating the highest degree of protection in cardiomyocytes (Fig. 6b, c). Furthermore, significant changes in protein markers were observed reflecting reduced senescence, including expression of γ-H2AX, LaminB1, P16, IL6, TNF-α (Suppl. Fig. 4), cell cycle suspension (Fig. 6f–i), and SA β-Gal staining (Fig. 6d, e), following pre-treatment with ZOL, CLO, or RIS.

### Global profiling of Zoledronate-binding proteins

**Fig. 7:**
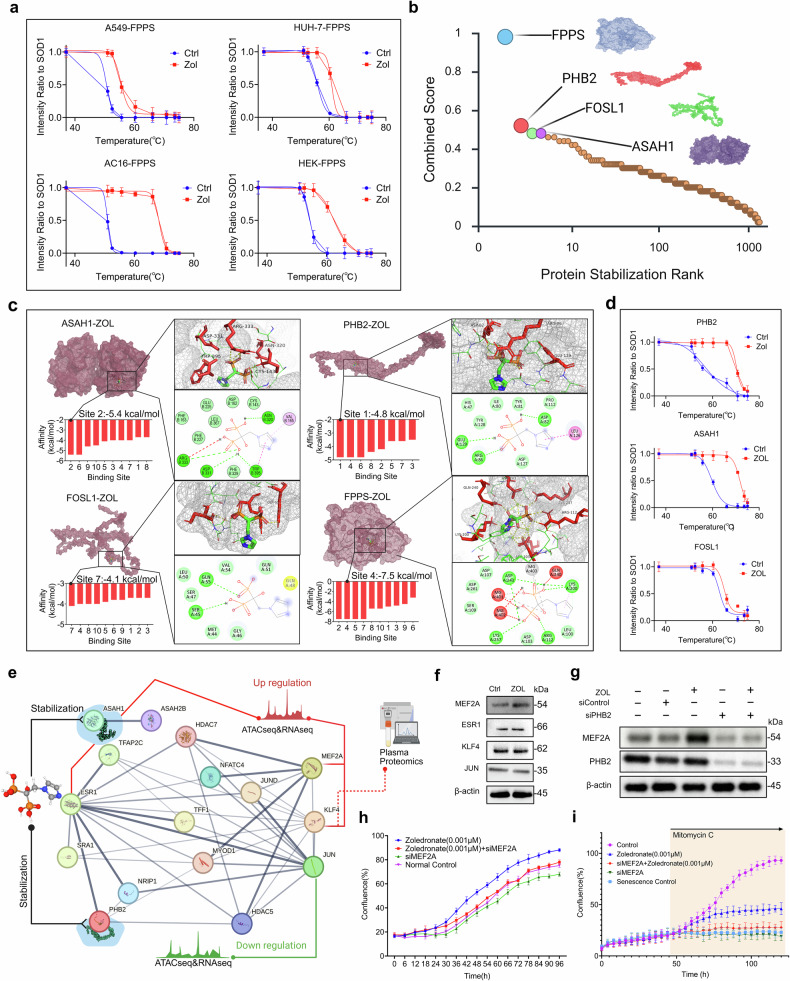
2D-thermal proteome profiling of Zoledronate binding targets. **a** FPPS thermal stability profiles following ZOL/vehicle ctrl treatment (A549, HEK293, AC16, HUH-7) relative to SOD1 (internal control). Detailed in Suppl. Table7 and Fig. 5. **b** ZOL stabilized proteins (AC-16) following 2D thermal profiling ranked by combined score. **c** The molecular docking results of Zoledronate and ASAH1, PHB2, FOSL1 and FPPS. The ranked binding sites with energy were shown as bar plot and the top-ranked binding pose for each protein were illustrated in surface and stick mode with labeled residuals and contacts. Both 3D and 2D illustration were made to illustrate ligand-receptor interactions. **d** CETSA**-**western blot validation of thermal profiling hits (ASAH1, PHB2, FOSL1, HMGA1). **e** Cross-omic analysis following ATAC-seq and RNA-seq of ZOL-treated (0.001 μM) AC-16 cells, downstream of prioritized CETSA targets (PHB2 and ASAH1). Further mapped to human proteome analysis following ZOL treatment (using STRING). **f** Immunoblotting of ZOL-induced protein changes (MEF2A, ESR1, KLF4 and JUN). **g** Immunoblotting of MEF2A and PHB2 protein levels in AC16 cells transfected with control siRNA (siCtrl) or PHB2 siRNA (siPHB2) and treated with zoledronate (ZOL) or vehicle. β-actin served as a loading control. **h** Time-course effects of MEF2A knockout on AC-16 cell growth ZOL pretreatment. **i** Time-course effects of MEF2A knockout on AC-16 cell growth following ZOL pretreatment and mitomycin C induction of senescence

Based on previous evidence indicating ZOL treatment favorably alters the plasma proteome of osteopenic patients, triggers beneficial transcriptomic changes at multiple organ sites in vivo, and promotes cell growth at low doses in multiple human cell types in vitro including human cardiomyocytes, we prioritized ZOL for 2D thermal profiling to investigate its proteome-wide target spectrum. This method measures protein stabilization at target saturating compound concentrations upon application of a heat gradient. First, we confirmed by western blot that the known ZOL target farnesyl pyrophosphate synthase (FPPS) was stabilized upon ZOL treatment over increasing temperatures (37–78 °C) in intact A549, HEK, AC16, and HUH7 cells. AC16 cardiomyocytes showed the most significant thermal stabilization effect across all four cell lines (ΔT_m_ = 17.38 °C) (Fig. 7a). Protein lysates of intact AC16 cardiomyocytes collected at increasing temperatures with different concentrations of ZOL (0-20 μM) were further analyzed using mass spectrometry and TP-MAP^101^ to identify proteins that might be positively or negatively (destabilized) altered by ZOL binding. Reassuringly, we were able to identify the cognate target FPPS, as the highest-scoring protein across 8397 identified proteins, following ZOL treatment (Fig. 7b and Suppl. Table 7). In addition, several other proteins (ASAH1, PHB2 and FOSL1) not associated with BPs, were also significantly stabilized following ZOL treatment (Suppl. Fig. 5). Computational molecular docking was performed on each of our top 4 protein targets, to calculate the most stable binding site and free energy, with accurate protein structures and visualization of BP binding (generated by AlphaFold). As anticipated, the binding free energy of the known BP target FPPS was lowest (−12.19 kcal/mol) followed by ASAH1 (−11.18 kcal/mol), PHB2 (−7.34 kcal/mol) and FOSL1 (−7.0 kcal/mol), indicating a relatively high binding affinity of ASAH1 and PHB2 (Fig. 7c). PHB2 is a multifunctional protein involved in various cellular processes such as mitochondrial function, cell cycle regulation, and apoptosis.^102^ ASAH1 encodes an enzyme involved in the metabolism of ceramides, which plays a role in various cellular processes, including cell signaling and apoptosis,^103^ whilst FOSL1 is a crucial component of the AP-1 transcription factor complex playing a key role in regulating cell proliferation, differentiation, and tumorigenesis.^104^ The direct target engagement was further confirmed by western blot based cellular thermal profiling (Fig. 7d) for PHB2, ASAH1, and FOSL1 (ΔT_m_ PHB2 = 12.15 °C, ΔT_m_ ASAH1 = 12.13 °C, ΔT_m_ FOSL1 = 2.09 °C), validating computational docking findings for PHB2 and FOSL1 as effective and novel BP-binding targets.

### Cross-omics identified PHB2-MEF2A axis in Zoledronate-induced cellular effects in cardiac tissue

To systematically explore the regulatory network associated with ZOL-stabilized protein targets, RNA- and ATAC-seq analysis were performed on AC-16 cardiomyoblasts with or without ZOL treatment (0.001 μM, 4 days) to identify changes in gene transcription and chromatin accessibility, respectively (supplementary Data. 2 and supplementary Data. 3). An expanded protein-protein interaction network (PPI), originating from PHB2 and ASAH1, was constructed using the STRING database. All factors related to these targets were mapped to our RNA-, ATAC-seq, and SomaScan proteomic datasets to identify genes or proteins common to all analyses and altered in human systems (in vitro and *vivo*) following exposure to ZOL. This integrated analysis indicated four potential regulators (ESR1, MEF2A, JUN, KLF4) downstream of identified protein targets of ZOL (Fig. 7e). Western blot analysis confirmed the sustained translational impact of MEF2A only, showing increased protein expression following ZOL treatment (Fig. 7f). MEF2A, myocyte enhancer factor 2a, is a transcription factor plays a central role in muscle development and cardiovascular biology^105^ and was found to suppress senescence in vascular endothelial cells.^106,107^ Similarly, our murine heart transcriptomic and plasma proteomic data showed MEF2A targets genes (Corin, Ablim1 and Nduf10) and related proteins (HMBS, BPGM and HIST2H2BE) were also upregulated following ZOL treatment (Suppl. Fig. 6), whilst PHB2 knockout in cardiomyoblast cells reduced MEF2A levels (Fig. 7g). To confirm the role of MEF2A in mediating the cellular effects of ZOL, MEF2A knockdown was also performed. Loss of MEF2A significantly reduced cell growth compared with vehicle-treated controls, indicating that MEF2A is essential for maintaining cardiac cell viability and consistent with previous findings. Following MEF2A silencing, the ZOL-induced growth-promoting effect was largely abolished (Fig. 7h). In the MMC-induced senescence model, MEF2A knockdown did not alter the baseline senescence response to MMC alone but markedly attenuated the senescence-protective effect of ZOL (Fig. 7i). Collectively, these findings identify the PHB2-MEF2A axis as a promising candidate pathway for ZOL-mediated cell growth and resistance to MMC-induced senescence.

## Discussion

While it remains clear that the calcium-binding property of BPs, along with their capacity to bind and inhibit FPPS in bone-resorbing osteoclasts^108^ constitutes the major effect and mechanism of this class of drugs in humans, this study highlights key areas where and how BP treatment elicits new effects. BP use varies across the globe, with trials primarily focused upon white female populations in developed regions such as the United States, United Kingdom, and New Zealand. Given the genetic and environmental variations influenced by gender, race, and geography, the impact of BPs on disease progression and mortality reduction could significantly differ across different demographics. Improved information on regions, races, and particularly male subjects, will be needed to form a complete picture of BP impact. However, a strong and clear pattern indicating significant benefit of BP treatment across multiple disorders has emerged, and where a single mechanism of action would be unlikely. In beginning to explore this polypharmacology of BPs, we have uncovered unique binding partners and downstream regulators that may collectively impact lifelong human health and prevent disease.

Treatment with ZOL in murine and human studies triggered significant genomic and proteomic changes respectively, in multiple tissues. This included alterations in immune cell regulation and changes to inflammatory factor profiles. In support of these findings, BPs have been shown to activate γ∆T-cell populations.^109^ This phenomenon may account for the acute phase response triggered by some BPs, that leads to flu-like symptoms following start of treatment.^110,111^ Female osteopenic patients treated with ZOL also upregulated circulating levels of proteins controlling autophagy, a natural process of waste disposal and recycling, that decreases with ageing, preventing the production of defective proteins. Autophagy is dependent upon effective endosomal/lysosomal activity to capture, internalize, and degrade defective proteins within an autophagosome.^112^ Our combined fluoro-labeled BPs and proteomic analysis tracked internalized BPs to these intracellular loci and confirmed elevated circulating levels of protein markers of lysosomal activity. In a similar approach to that adopted in our study, Yu, Surface, and colleagues identified new BP transporter molecules using CRISPR-mediated genomic analysis following ZOL-treatment. Through a SLC37A3-ATRAID complex, bound BPs transported through the cytosol are released at lysosomal sites^19^ supporting a role for BPs in ageing-linked autophagy control and highlighting the possibility of further BP binding targets.

Micromolar levels of BPs have been reported to promote tumour cell apoptosis through activation of autophagic cell death in a range of cancers including prostate, breast, cervical, colon, and osteosarcoma. This is suggested to occur via reduced activity of the mevalonate pathway enzyme, geranylgeranyl diphosphate (GGDP). However, this could not be rescued through re-addition of mevalonate pathway metabolites or use of other pathway inhibitors, suggesting BP action is only partly mediated by mevalonate pathway inhibition and where other mechanisms (e.g., inhibition of mTOR signaling) will play a role.^113–118^ Modification of mTOR signaling by BPs has also been linked to survival in mesenchymal stem cells, where ZOL conferred protective effects against DNA-damage,^119^ and improved survival in Drosophila.^120^ This might also account for rejuvenation-like effects seen in our animal studies where ZOL-treatment in aged mice induced a tissue-specific (heart, liver, lung) local transcriptomic and cell type proportion change, reflecting that of young animals. Non-proliferative senescent cells form and accumulate in tissues with increasing age. These cells corrupt the local environment by secreting a toxic cocktail including inflammatory factors and matrix-degrading enzymes,^121^ whilst removal of senescent cells or preventing their formation in the first instance, improves tissue function.^121^ Collectively, our work shows low dose BP-treatment prevents the induction of DNA damage-related senescence and products (SASP), exhibiting senomodifying effects across multiple cell types.^23^ Although nanomolar-level BP exposure produced beneficial effects on cell growth and senescence prevention in our in vitro model, such low and sustained concentrations cannot be directly or precisely achieved through standard clinical zoledronate infusion, owing to rapid skeletal sequestration and renal clearance. As a result, non-skeletal tissues are likely exposed to only indirect, delayed, and poorly controlled BP levels following systemic administration. These pharmacokinetic constraints present a major barrier to direct clinical application of low-dose BP in non-skeletal tissues. Consequently, the development of alternative delivery strategies capable of providing controlled, long-term, low-level exposure may be required to harness these benefits in vivo.

Using AlphaFold, computational molecular docking alongside multi-omics analysis we have revealed new targets of BP action influencing protection against the induction of senescence, and potential onset of an ageing phenotype through binding of PHB2 and activation of MEF2A. The prohibitin family (PHB) are highly conserved, widely expressed pleiotropic proteins implicated in a variety of cellular functions including proliferation, apoptosis, transcription and tumor suppression. Importantly, PHB2 is a mitochondrial scaffold protein linked to increased lifespan brought about by improved mitochondrial stability,^122,123^ and similar to BPs, protects against cardiovascular disease and neurodegeneration.^124–126^ Similarly, the expression of the MEF2A transcription factor declines in ageing tissues, whilst MEF2A activation may improve skeletal muscle function in aged animals, cognitive decline in neurodegenerative models, and endothelial cell function in cardiovascular disease.^127–129^ Furthermore, mutations in MEF2A have been reported in families with CVD.^130,131^ In support of our findings, MEF2A deletion led to increased senescence in coronary artery endothelial cells^106^ suggesting that MEF2A activation following BP treatment is protective in this system. Also, ASAH1 is a lysosomal enzyme involved in sphingolipid metabolism and fluoro-labeled BPs were found co-localized within lysosomes and endosomes. These observations suggest that BPs may influence intracellular homeostasis through a combination of direct lysosomal effects (ASAH1) and indirect modulation of mitochondrial and nuclear pathways (PHB2, MEF2A), without requiring substantial drug accumulation within these organelles.

As the human proteomic analyses were derived exclusively from female participants enrolled in randomized controlled trials, and in vivo experiments were conducted in female mice, reflecting the demographic focus of long-term osteoporosis studies and available animal models, the impact on male subjects remains speculative. In contrast, the in vitro experiments incorporated cell lines of both female and male origin and were designed to interrogate shared cellular mechanisms of BP action. No apparent differences were observed based on sex of origin, with studies incorporating large male clinical cohorts and sex-stratified animal models needed to assess potential sex-specific effects. Similarly, the high bone affinity and rapid systemic clearance of BPs, mean detecting BP accumulation in non-skeletal tissues remains technically challenging, and where in vitro internalization data may not fully replicate the complex in vivo biodistribution of BPs. Another important consideration arising from our findings is the observation that low-dose BPs elicited pro-growth effects, which may stimulate normal and abnormal cells, highlighting a potential risk. However, these effects were observed in highly reductionist systems that lack critical in vivo constraints, including immune surveillance, stromal interactions, and tissue-level homeostatic mechanisms that actively limit the expansion of damaged or aberrant cells. Moreover, bone-invasive malignancies like myeloma and prostate cancer cells may encounter relatively high local BP concentrations that favor cytotoxic effects. Until now, there is no solid clinical evidence confirming the role of BP on cancer progression. Furthermore, our mechanistic investigation focused on the cardiac response to ZOL due to its more robust tissue-level sensitivity and superior stabilization of FDPS compared to other N-BPs. It is possible that PHB2 and ASAH1 are not primary targets for RIS or ALN. Moreover, the binding profile of ZOL may vary across non-cardiac cell types, driven by the inherent heterogeneity of intracellular proteomes.

**Fig. 8:**
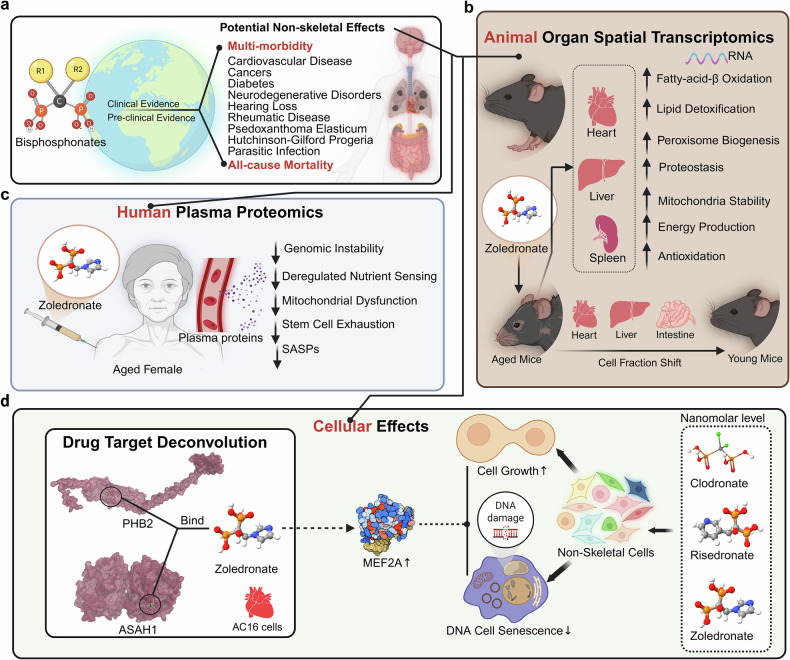
Graphical summary - Extraskeletal geroprotective effects of bisphosphonates. **a** Global evidence from both clinical and pre-clinical studies illustrates the potential non-skeletal effects of bisphosphonates on multimorbidity and mortality. **b** Zoledronate alters murine transcriptomic profile across multiple sites including upregulation of fatty acid β-oxidation, mitochondrial stability, and antioxidation, and shifts cell populations toward more youthful states. **c** Zoledronate reshapes human plasma proteome, downregulating proteins linked to genomic instability, proteostasis loss, mitochondrial dysfunction, stem cell exhaustion, and SASP. **d** Bisphosphonates enhance non-skeletal cell growth at low doses and prevent senescence. Particularly in heart cells, where Zoledronate binds to previously unrecognized protein targets (PHB2, ASAH1), upregulates MEF2A

In summary, our research presents a comprehensive analysis of BP effects beyond their traditional use in preserving bone health, where their positive impact on plasma proteomics of ageing pathways cellular senescence, and rejuvenation of organ-specific cell populations offers new therapeutic possibilities. These findings expand our current understanding of the operational mechanisms of BPs within human cells, and impact upon local tissues in vivo and systemic effects in humans (Fig. 8). These suggest both FPPS-dependent and new independent mechanisms through which BPs might confer beneficial effects in ageing-related disorders.

## Materials and Method

### Disease based illustration of novel benefits from bisphosphonates

A systematic approach was employed to gather clinical and pre-clinical studies reporting non-osteoporotic benefits of bisphosphonates from major public medical databases, including PubMed, Scopus, and Web of Science. The search strategy focused on keywords related to bisphosphonates (“Alendronate”, “Clodronate”, “Etidronate”, “Risedronate”, “Minodronate”, “Ibandronate”, “Zoledronate”, “Pamidronate”, “Neridronate”, “Tiludronate”, “Incadronate” and “bisphosphonates”) and their broad effects on human disease (including “cardiovascular disease”, “neurodegenerative disease”, “cancer”, “tumour”, “immune disease”, “infection”, “endocrine disease” and “rare disease”). Studies with a focus on both osteoporosis and other non-osteoporotic diseases were also included. To visually demonstrate the data, a disease-based illustration was created (BioRender.com). For each disease, the corresponding bisphosphonate was indicated along with coloured markers representing the source (clinical evidence-green dots; pre-clinical evidence-black dots; working mechanisms of bisphosphonates related to FPPS inhibition/mevalonate pathway-yellow dots; other mechanisms-red dots).

### Geographical heatmap analysis and illustration

A systematic approach was employed to gather randomized controlled trials (RCTs) describing effects of bisphosphonates and non-cancer mortality from public medical databases, including PubMed, Scopus, Cochrane Library, and Web of Science. The search strategy focused on keywords related to bisphosphonates (“Alendronate”, “Clodronate”, “Etidronate”, “Risedronate”, “Minodronate”, “Ibandronate”, “Zoledronate”, “Pamidronate”, “Neridronate”, “Tiludronate”, “Incadronate” and “bisphosphonates”) and death (including “mortality”, “lifespan”, “ageing”, or “senescence”). For all RCTs identified, only studies relating to the mortality-reducing effects (both statistically significant and insignificant) of bisphosphonates were collected for further analysis and data related to the location, number, sex and race of patients, and types of bisphosphonates used are described. A geographical heatmap of enrolled RCTs was created (Datawrapper), with colour intensity indicating patient number, dotted line indicating drug type. The gender ratio was illustrated as pie charts and the percentage of patients tested for specific drug types was illustrated using dot plots. Both figures were created through GraphPad Prism version 9.0 (GraphPad Software, USA).

### Human plasma proteomics and analysis

Plasma samples were collected from 36 osteopenic female patients aged over 65 years who participated in a RCT conducted in New Zealand.^96^ Samples for each patient were obtained at three stages (baseline-prior to treatment, 18 mths post-treatment, 36 mths-post treatment) after initiating a 5 mg Zoledronate injection regimen. Following blood clotting and centrifugation at 1,500 × g for 10 min, plasma was carefully extracted, aliquoted and stored at -80°C. Proteomic profiling was performed using the SomaScan® platform (SomaLogic, USA). The raw protein expression data were normalized using proprietary normalization techniques developed by SomaLogic, involving scaling and log transformation to correct batch effects. Subsequent downstream analysis was performed using R and R studio (version 2.8.2) with specific Bioconductor (3.19) and CRAN (4.4.0) packages. Differential protein expression analysis was conducted using the limma package and volcano plot analyses of APPs were performed with EnhancedVolcano. Mapping analysis of SASP and possible ageing-related APPs was conducted using the SenoMayo gene set^132^ and The Human Ageing Genomic Resources (HAGR)^133^ with four major divisions (GenAge, GenDR, LongevityMap and CellAge) focusing on genes related to human ageing, dietary restriction, genetic variants linked to longevity and senescence, respectively.^134^ Bar plots were generated from Biorender. Raw data has been stored in figshare (10.6084/m9.figshare.31136839).

### Animal organ histology and spatial transcriptomics

The in vivo study was conducted using 18 female C57BL/6 N mice, including six 2-month-old young mice and twelve 22-month-old mice (at Mayo Clinic, MN, USA). Six 2-month-old mice were treated with vehicle control, six 22-month-old mice were treated with vehicle control, six 22-month-old mice were treated with 125 μg/kg Zoledronate for 2 months. After treatment, seven organ tissues of interest, including liver, kidney, heart, spleen, lung, intestine and pancreas, were harvested to assess potential transcriptomic changes triggered by ZOL. Tissues were fixed in 4% paraformaldehyde, embedded in paraffin, sectioned at 5μm thickness, and subjected to H&E. Tissue cores (1 mm diam) were selected from corresponding areas within each organ and a total of four tissue microarrays (TMA) were assembled. After tempering, the TMAs were sectioned at 5 μm thickness and immunofluorescence staining performed with PanCK, CD45 and SYTO13 to mark epithelial cells, leukocytes and nuclei respectively. The stained sections were visualized, and ROIs selected based on typical tissue types across different treatment groups (Suppl. Table 4), followed by transcriptomic profiling of ROIs under Nanostring GeoMx® Digital Spatial Profiler (Bruker, USA). FASTQ files were processed using the NanoString GeoMx DSP Control Centre (version 3.1.0.222) and subjected to quality control (QC) checks, including a minimum threshold of 1000 raw reads, sequencing saturation exceeding 50%, and more than 80% of reads being aligned, stitched, and trimmed. Regions of interest (ROIs) with fewer than 10% of genes expressed were excluded. Genes undetected in more than 10% of ROIs were removed and raw counts were normalised using Q3 normalisation. DESeq2 was used to identify differentially expressed genes between the treatment groups (aged control vs. Zoledronate-treated) within each organ. UMAP analysis were conducted using the Seurat package to visualize overall sample heterogeneity. The Biorender was used to create heatmaps illustrating the expression patterns of ageing-related DEGs across the different organs. CybersortX tool^135^ was employed for cell population deconvolution analysis from bulk transcriptomic data with Tabula Muris^136^ database-derived gene signature for each organ. Raw data has been stored in figshare (10.6084/m9.figshare.31136839).

### Cell culture, bisphosphonates treatment and senescence induction

Ten non-skeletal cell lines were cultured under conditions tailored to each cell type. These included HL-1 (mouse cardiac muscle cell line, Sigma-Aldrich, cat.no SCC065), C2C12 (mouse myoblast, ATCC, cat.no CRL-1772), THP-1 (human monocyte, ATCC, cat.no TIB-202), HUH-7 (human hepatoma cells, Cytion, cat.no 300156), PNT-2 (human prostate cell, ATCC, cat.no 95012613), HEK (human embryonic kidney cells, ATCC, cat.no CRL-1573), A549 (human alveolar basal epithelial cells, ATCC, cat.no CCL-185), AC-16 (human cardiomyocyte, Sigma-Aldrich, cat.no SCC109), BMSCs (bone marrow mesenchymal stromal cells, Sigma-Aldrich, cat.no SCC034), and HCMVECs (human cardiac microvascular endothelial cells, PromoCell, cat.no C-12285). Each cell line was maintained in its respective medium as follows: HL-1 in Claycomb medium with 10% FBS, 2 mM L-glutamine, and 1% penicillin-streptomycin; C2C12, HUH-7, HEK, and A549 in DMEM with 10% FBS and 1% penicillin-streptomycin; THP-1 and PNT-2 in RPMI-1640 medium with 10% FBS and 1% penicillin-streptomycin; AC-16 in F-12 medium with 12.5% FBS, 1% L-glutamine, and 1% penicillin-streptomycin; BMSCs in α-MEM with 10% FBS, 1% L-glutamine, and 1% penicillin-streptomycin; and HCMVECs in Endothelial Cell Growth Medium (EGM, R&D Systems, cat.no C-12285) with manufacturer-provided supplements. To induce DNA damage-related senescence, cells were treated with a dose panel of mitomycin C (Sigma, cat.no M4287) ranging from 0 to 0.05 μg/ml for up to 4 days, to identify the optimal dose for triggering senescence phenotype. For bisphosphonate treatment, cells were first cultured to 20% confluence and treated with low (0.001 μM), middle (0.1 μM), and high (10 μM) doses of seven bisphosphonates (Alendronate, Clodronate, Etidronate Lidadronate (IG9402), Risedronate, OX14 (OX14 (4-[N-(3-pyridylsulfonyl)-3-aminopropyl]-1-hydroxy-1,1-bisphosphonic acid and Zoledronate) for 4 days or pretreated with various doses of the same bisphosphonates for 2 days, followed by the addition of 0.05 μg/ml mitomycin C to induce senescence. All experiments were conducted in triplicate to ensure reproducibility and statistical significance.

### Confocal microscopy for uptake and subcellular distribution of bisphosphonates

Cells were cultured under optimal conditions and seeded onto chambered coverslips (Ibidi, cat.no 80826). For bisphosphonate internalization studies, cells were treated with fluorescently labelled compounds 5(6)-ROX-RIS (0.1 μM, BioVinc LLC, cat.no BV150101), 5-FAM-ZOL (0.1 μM, BioVinc LLC, cat.no BV121001), or ATF647-ZOL (0.1 μM, BioVinc LLC, cat.no BV501001) for 48 hours. For co-localization analysis, cell organelles were labelled using the CellLight system (ThermoFisher) with specific markers: Mitochondria-GFP (cat.no: C10600), Lysosomes-GFP (cat.no: C10597), Endosomes-GFP (cat.no: C10586), Golgi-GFP (cat.no: C10592), and Peroxisomes-GFP (cat.no: C10604) following fluorescent bisphosphonate treatment, for 2 days. Additionally, cytoskeletal components were stained with Phalloidin-488 (Thermo Fisher Scientific, cat.no A12379) or Phalloidin-594 (Thermo Fisher Scientific, cat.no A12381) to match the combination of different wavelengths of fluorescence 20 min ahead of observation. After staining, cells were fixed and nuclei counterstained with DAPI (Thermo Fisher Scientific, cat.no D1306), preparing them for confocal microscopy with a Zeiss LSM 710 confocal microscope (Carl Zeiss AG) equipped with Zen Black software (version 2.3). Z-stack image acquisition was utilized to visualize the 3D location of bisphosphonates and organelles. Image analysis software, Zen Blue (version 3.1, Carl Zeiss AG) and Fiji image J^137^ with JACoP plugin, was used to process the Z-stack images, calculating colocalization coefficients (Pearson’s coefficients) to quantify the overlap between the fluorescent signals of bisphosphonates and organelles.

### Live cell imaging

Cell growth dynamics in response to bisphosphonate treatment or mitomycin C induction were monitored using a live-cell analytical system (Incucyte® S3 Live-Cell Analysis System, Sartorius). Real-time imaging was conducted automatically, with representative images captured every four hours. IncuCyte® software (version 2022 A, Sartorius) was employed to quantify cell confluence ratio using advanced AI algorithms. For each treatment group, at least three replicate wells of cells were utilized. In each well, images from nine different vision fields were captured to calculate the average confluence ratio, ensuring statistical robustness and accuracy in the analysis.

### SA-β-GAL staining

Senescence associated β-gal was detected using the Senescence β-Galactosidase Staining Kit (Cell Signalling Technology, cat.no 9860) according to the manufacturer’s instructions. Briefly, cells were seeded on 96 well plates with corresponding culture medium and drug treatments. Post-treatment, cells were fixed, washed with sterile PBS, and incubated overnight in staining solution at 37°C in a CO_2_ free incubator. The results are shown as a ratio of β-gal positive (blue colour) cell number and total cell number.

### Western blot

Treated cells were lysed using ice-cold RIPA buffer and protein concentration confirmed using the BCA assay (Thermo Fisher, cat.no 23225). Western blotting was performed with primary antibody of typical senescence markers including p16^INK4a^, p21^WAF1/CIP1^, Phospho-Histone H2A.X, LaminB1, HMGB1, IL-6, and TNF-α (Cell Signalling Technology, cat.no 56062) at 1:2000 overnight. Protein expression levels are expressed relative to β-actin expression. Three technical replicates were performed. Data analysis was conducted with Image J and GraphPad Prism version 8.0.

### Cell cycle analysis

Following treatment, cells were prepared for flow cytometry using PI (500 µg/ml) on BD LSRFortessa™ cell analyzer (BD Biosciences). Cell cycle distribution was analysed by ModfitLT 5 (Verity Software House).

### Molecular docking

After CETSA-MS, the binding capability of the top 4 potential protein targets with Zoledronate was also assessed computationally by AMDock v1.5.2 tools^138^ (https://github.com/Valdes-Tresanco-MS/AMDock-win), which is a GUI platform integrating Autodock4^139^ and PyMOL (The PyMOL Molecular Graphics System, Version 3.0 Schrödinger, LLC.) for both docking analysis and visualization (details in supplementary data).

### ATAC-seq

AC-16 cells were treated with low dose (0.001 μM) of Alendronate, Clodronate, Etidronate, Lidadronate, OX14, Risedronate, or Zoledronate for 4 days. Following treatment, the total DNA was extracted for ATAC-seq library preparation using the Active Motif ATAC-Seq Kit (Active Motif, cat.no 53150) according to the manufacturer’s instructions. ATAC-seq libraries were sequenced (NextSeq 500 sequencer, Illumina) using the mid-output kit (Illumina, cat.no 20024904) to generate 75 bp paired-end reads. Raw sequencing data were processed using a bioinformatics pipeline on a Linux system. This included quality control with FastQC (Version 0.11.9), trimming of adapter sequences with Trim Galore (Version 0.6.6), and alignment to the human reference genome (hg38) using Bowtie2 (Version 2.4.2). Peaks were called using MACS2 (Version 2.2.7.1), and significant peaks were annotated with ChIPseeker (Version 1.26.2) in R Studio. Raw datasets have been stored in figshare (10.6084/m9.figshare.31136839).

### RNA-seq

AC-16 cells were treated with low dose (0.001 μM) Clodronate, Risedronate or Zoledronate for 4 days. After treatment, total RNA was extracted using the Aurum™ Total RNA Mini Kit (Bio-Rad, cat.no 7326820) according to the manufacturer’s instructions. RNA integrity and concentration were assessed using an Agilent 2100 Bioanalyzer (Agilent Technologies). RNA-seq libraries were prepared using the TruSeq Stranded Total RNA with Ribo-Zero Globin kit (Illumina, cat.no 20020598). The libraries were sequenced and raw data processed as ATAC-seq. Reads were aligned to the human reference genome (hg38) using STAR aligner (Version 2.7.8a). Gene expression quantification was performed with featureCounts (Version 2.0.1). Downstream analysis of the processed files was conducted in R Studio. Differential gene expression analysis was performed using DESeq2. Gene Ontology (GO) pathway analysis was conducted using the ShinyGo platform to identify significantly enriched biological processes. Venn plots were generated using the SRplot tool to compare differentially expressed genes across different treatment conditions. Ranking analysis was conducted in Microsoft Excel, and the results were visualized as heatmaps using TBtools. Raw data has been stored in figshare (10.6084/m9.figshare.31136839).

### CETSA-WB and 2D Thermal Profiling

FPPS was applied as the known target of ZOL to select responsive cell types (HUH-7, HEK, A549, AC-16 cells). Following treatment, cell lysates were subjected to a temperature range (37 °C, 51 °C, 52.5 °C, 55.7 °C, 60.3 °C, 66.1 °C, 70.8 °C, 73.6 °C, 75 °C, and 78 °C) for 3 minutes to induce protein denaturation. Western blot was performed as described and using primary antibodies specific to human FPPS (Thermo Fisher, cat.no MA5-44777) at 1:1000 dilution (SOD1 (Cell Signaling Technology, cat.no 2770) was applied as internal control). The intensity of FPPS protein expression was quantified using Fiji ImageJ (v2.9.0) and analyzed using a Boltzmann sigmoidal equation to determine melting temperature (Tm). Further proteins assessed were: ASAH1 (Cell Signaling Technology, cat.no 13819), PHB2 (Cell Signaling Technology, cat.no 14085), FOSL1 (Cell Signaling Technology, cat.no 5281), and HMGA1 (Cell Signaling Technology, cat.no 7777) (all at 1:1000 dilution). For 2D thermal profiling, AC-16 cells were cultured as described with increasing concentrations of ZOL (0 μM, 0.2 μM, 2 μM, 5 μM, 20 μM) for 40 min. Cells were subjected to a thermal gradient (as described above), and protein concentrations were determined by BCA assay. Protein lysates were processed via S-Trap trypsin digestion and TMT10plex labeling, followed by high-pH fractionation and analysis on an Orbitrap Ascend platform using an RTS-SPS-MS3 quantification strategy (details seen in supplementary data). Raw mass spectrometry data were processed using Proteome Discoverer for protein identification and quantification. Statistical analysis was performed using the Thermal Profiling Meltome Analysis Program (TP-MAP) software package (https://gitlab.com/ChemBioHub/tpmap).^86^ The combined score was applied to evaluate the rank of stabilized (> 0) and destabilized (< 0) targets. Raw datastets have been stored in figshare (10.6084/m9.figshare.31136839).

### Cross-omic analysis and validation

Cross-omic analysis was performed, integrating data from 2D thermal profiling, ATAC-seq, RNA-seq, and SomaScan proteomic studies. The analysis utilized STRING database identifying downstream interactive proteins. Overlap analysis highlighted ageing-related factors, subjected to further validation by western blot: MEF2A (Cell Signaling Technology, cat.no 5030), c-JUN (Abcam, cat.no ab32137), KLF4 (Cell Signaling Technology, cat.no 12173), ESR1 (Cell Signaling Technology, cat.no 8644), PHB2, ASAH1 and FOSL1 (at 1:1000 dilution). For knockout of MEF2A and PHB2, AC-16 cells were transfected with ON-TARGETplus SMARTpool siRNA targeting human MEF2A or PHB2 (Dharmacon, Horizon Discovery) or a non-targeting control siRNA using Lipofectamine RNAiMAX (Thermo Fisher Scientific) according to the manufacturer’s instructions. Knockdown efficiency was confirmed by immunoblotting, showing > 70% reduction of MEF2A and PHB2 expression relative to non-targeting controls. After bisphosphonates treatment for 72 h, cells were treated with siRNA again to maintain the knockout effects.

### Graphic illustration

Graphical schematics in Fig. 1b, Fig. 2a, Fig. 3a, and Fig. 8 were created using the online scientific illustration platform BioRender (BioRender.com). Figure components were manually assembled and customized within the web-based BioRender environment and exported for manuscript preparation.

### Statistical analysis

Data are presented as mean ± standard deviation (SD). Comparisons between two groups are performed using Student’s t-test, while one-way ANOVA followed by a post-hoc test is used for multiple comparisons. A *p*-value of less than 0.05 is considered statistically significant.

## Supplementary information

Supplementary Materials

Data.S1

Data.S2

Data.S3

## Data Availability

All data and figures required to support the conclusions of this study are provided in the article and supplementary materials. The datasets of human plasma proteomics, animal organ transcriptomics, cellular RNAseq, ATACseq, and 2D-Thermal proteomics profiling results are stored in figshare (10.6084/m9.figshare.31136839). Additional supporting data are available from the corresponding author upon reasonable request.
