# Supplementary material for: Multiomic profiling of responses to clinical and novel bisphosphonates reveals extraskeletal effects on ageing related signatures
Source: Signal Transduct Target Ther. 2026 Jul 15;11:277. doi: 10.1038/s41392-026-02799-x (PMC13370019; doi:10.1038/s41392-026-02799-x)
Supplement: Supplementary file 1 — Supplementary Materials [file 41392_2026_2799_MOESM1_ESM.docx]

Supplementary Materials for

**Multiomic Profiling of Responses to Clinical and Novel Bisphosphonates Reveals Extraskeletal Effects on Ageing Related Signatures**

Jinsen Lu^1^, Srinivasa Rao Rao^2^, Helen Knowles^1^, Haoqun Zhan^1^, Beatriz Gamez^2^, Mingyu Qin^3^, Eleanor Platt^4^, Lucy R. Frost^4^, Tiffany-Jayne Allen^4^, Gayle Marshall^4^, Kilian V.M. Huber^5,6^, Ludwig G. Bauer^5,6^, Iolanda Vendrell^5,6^, Darragh P O'Brien^5,6^, Benedikt Kessler^5,6^, Anne Horne^7^, Ian R Reid^7^, Chas Bountra^6^, James L Kirkland^8^, Sundeep Khosla^9^, F Hal Ebetino^10^, Emilio Roldan^11^, R Graham G Russell^1,12^, James R Edwards^1*^

Correspondence to: James.Edwards@ndorms.ox.ac.uk

**This PDF file includes:**

Additional Materials and Methods

Supplementary Figures 1 to 6

Supplementary Tables 1 to 7

Captions for Data S1 to S3

**Other Supplementary Materials for this manuscript include the following:**

Data S1 to S3 [Data.S1 Differentially expressed plasma proteins at 18 and 36 months post-treatment of Zoledronate; Data.S2 Differential gene expression analysis results of AC16 cells treated with zoledronate versus control; Data.S3 Differential ATAC-seq peak analysis of AC16 cells treated with zoledronate versus control.]

Additional Materials and Methods

Molecular docking and visualization

After CETSA-MS, the binding capability of the top 4 potential protein targets with Zoledronate was also assessed computationally by AMDock v1.5.2 tools^1^ (https://github.com/Valdes-Tresanco-MS/AMDock-win), which is a GUI platform integrating Autodock4^2^ and PyMOL (The PyMOL Molecular Graphics System, Version 3.0 Schrödinger, LLC.) for both docking analysis and visualization. Briefly, the PDB files of FDPS and ASAH1 were downloaded from RCSB PDB database (https://www.rcsb.org) while the PDB files of PHB2 and FOSL1 were generated and downloaded from AlphaFold protein structure database (https://alphafold.ebi.ac.uk). The SDF file of Zoledronate was downloaded from PubChem and transformed to PDB format using Open Babel. Then both proteins and Zoledronate were processed in ADT4^2^ to filter unwanted components, add polar hydrogen and assign Gasteiger charges. For those proteins, a grid box was set to cover the whole protein structure for blind docking analysis. Then the processed PDB files of both ligand and receptor were set as input in AMDock for docking analysis with Autodock4 and results of top docking poses and binding free energy were listed. The binding affinity of all poses was illustrated as a bar graph of free energy using GraphPad Prism 8. Protein-ligand docking visualization was performed using PyMOL. The target protein structure and its binding interface with zoledronate were visualized, with the protein displayed in both surface and wireframe representations, while the ligand was rendered in stick mode to highlight its orientation and interactions. For detailed 3D inspection, all residues involved in ligand binding and hydrogen bonding were labeled and displayed with 50% transparent surface representation to enhance visibility of the binding pocket. The PDB files of the target protein, docked ligand pose, and polar contacts were then exported and imported into Discovery Studio (Biovia, San Diego), where a 2D interaction diagram was generated to illustrate key ligand-receptor interactions such as hydrogen bonds and hydrophobic contacts.

CETSA-WB and 2D Thermal Profiling

FPPS was applied as the known target of ZOL to select responsive cell types (HUH-7, HEK, A549, AC-16 cells). Following treatment, cell lysates were subjected to a temperature range (37°C, 51°C, 52.5°C, 55.7°C, 60.3°C, 66.1°C, 70.8°C, 73.6°C, 75°C, and 78°C) for 3 minutes to induce protein denaturation. Western blot was performed as described and using primary antibodies specific to human FPPS (Thermo Fisher, cat.no MA5-44777) at 1:1000 dilution (SOD1 (Cell Signaling Technology, cat.no 2770) was applied as internal control). The intensity of FPPS protein expression was quantified using Fiji ImageJ (v2.9.0) and analyzed using a Boltzmann sigmoidal equation to determine melting temperature (Tm). Further proteins assessed were: ASAH1 (Cell Signaling Technology, cat.no 13819), PHB2 (Cell Signaling Technology, cat.no 14085), FOSL1 (Cell Signaling Technology, cat.no 5281), and HMGA1 (Cell Signaling Technology, cat.no 7777)(all at 1:1000 dilution). For 2D thermal profiling, AC-16 cells were cultured as described with increasing concentrations of ZOL (0 μM, 0.2 μM, 2 μM, 5 μM, 20 μM) for 40 minutes. Cells were subjected to a thermal gradient (as described above), and protein concentrations determined by BCA assay. For each sample, 100 μg of protein lysate was mixed with a final concentration of 5% SDS, reduced with 20 mM DTT for 30 min, and alkylated with 40 mM IAA for 30 min in the dark. Proteins were then digested with 2 μg trypsin (cat. No. V5111, Promega) for 20 h using S-Trap 96-well plates, following the manufacturer’s instructions (Protifi). Peptides were eluted and dried in a vacuum centrifuge. After resuspension in 100 μL of 100 mM TEAB, peptides were labeled with TMT mass tags according to the manufacturer’s instructions (Thermo Fisher). A single TMT10plex (Thermo Fisher, cat. no. 90110) was used to label two sets of 10 samples, each containing 100 μg of peptide. Labeled samples were pooled and frozen at -80°C. Pooled samples were desalted using Sep-Pak Plus C18 cartridges (Waters, cat. no. WAT020515) following the manufacturer’s instructions and dried. Peptides were resuspended in a buffer (2% ACN, 0.1% FA in mass spectrometry-grade H_2_O) and subjected to high-pH offline reverse-phase pre-fractionation using a Dionex Ultimate 3000 system (Thermo Scientific) on a C18 column (XBridge BEH C18, 2.5 μm, 3x150 mm, Waters) at a flow rate of 0.2 mL/min. Buffer A (100% H_2_O, pH 10) and buffer B (90% ACN, pH 10) were prepared with mass spectrometry-grade H_2_O, and the pH was adjusted using 17% NH_4_OH. Peptides were separated using a 100-minute gradient: 0–12 minutes, 0.2% B; 12–72 minutes, 2–45% B; 72–80 minutes, 45–95% B; 80–90 minutes, 95% B; 90–92 minutes, 95–2% B; 92–100 minutes, 2% B. A total of 50 fractions were collected by switching vials every 2 minutes, which were concatenated across the gradient to yield 10 samples before drying. Then the peptides were reconstituted in 0.1% formic acid prior to analysis using a high-resolution mass spectrometer (Thermo Fisher Scientific Orbitrap Ascend) coupled to a Vanquish Neo UHPLC system (Thermo Fisher Scientific, operated in Trap and Elute mode) equipped with a PepMap Neo trap (C18, 5μm, 300μm x 5mm, set up withback flash wash) and EASY-SPRAY PepMapNeo column (50cmx75μm, 1500bar). Peptides were separated using a 75 min gradient, going from 3 to 20% B (80% acetonitrile, 0.1% Formic acid) in 40 minutes, to 35 % B in 20 minutes, to 99% B in 1 min and kept at 99% B for an additional 14 minutes with a flow rate of 300nl/min. Peptides were analysed using the Real Time Search (RST) – MS3 method. Spectra were acquired in the Orbitrap Ascend using the Real time search (RST) combined with synchronous precursor selection (SPS) and MS3 reporter ion quantitation. Briefly, MS1 spectra were acquired in the Orbitrap mass analyser at 120K resolution with a scan range from 400 to 1600 m/z, normalized AGC target at 100%, RF at 30% and a maximum injection time of 251msec. MS2 spectra were acquired in the Ion trap (rapid scan mode) with a Quad isolation window of 0.7 m/z, normalized 100% AGC target, maximum injection time of 35msec with HCD activation and collision energy of 38%. Liner Ion trap spectra were subjected to the RTS using the Human Uniprot Swissprot reviewed fasta file (downloaded 20220222, 20386 sequences). Trypsin was chosen as proteolytic enzyme, carboamidomethylation (C) and TMTplex (Kn) were set as fixed modifications and Oxidation (M) as variable modification. Maximum missed cleavages were set at 1 and maximum variable mods per peptide was set to 2. TMT-SPS-MS3 mode was selected with a maximum search time of 100 msec, scoring threshold was set to 1.4Xcorr, 0.1dCn and 10ppm tolerance. Spectra were filtered using the Precursor selection range filter of 400 -1600 m/z and isobaric tag loss set to TMT. Precursors identified via RTS were isolated using the quadrupole (2 m/z window) and ions were collected for a maximum injection time of 105msec and normalized AGC of 200%. Synchronous Precursor Selection (SPS) was set to 10. Isolated fragments were fragmented in the HCD cell with 65% collision energy and MS3 spectra were acquired in the orbitrap at 45K resolution with a scan range of 100 – 500 m/z. The total duty cycle was set to 2.5 sec. Raw mass spectrometry data were processed using Proteome Discoverer for protein identification and quantification. Statistical analysis was performed using the Thermal Profiling Meltome Analysis Program (TP-MAP) software package (https://gitlab.com/ChemBioHub/tpmap)^3^. The combined score was applied to evaluate the rank of stabilized (>0) and destabilized (<0) targets.

Supplementary Figure 1. Tissue specificity of significantly altered plasma proteins in female patients.

Following differential analysis of plasma proteins from female patients before and after 18 months of zoledronate treatment, proteins with |logFC| > 0.5 were used as the assessment input. Tissue specificity analysis was performed using the TissueEnrich tool based on reference expression data from the Human Protein Atlas. Results are presented as foldchange bar plots indicating enrichment across tissues.


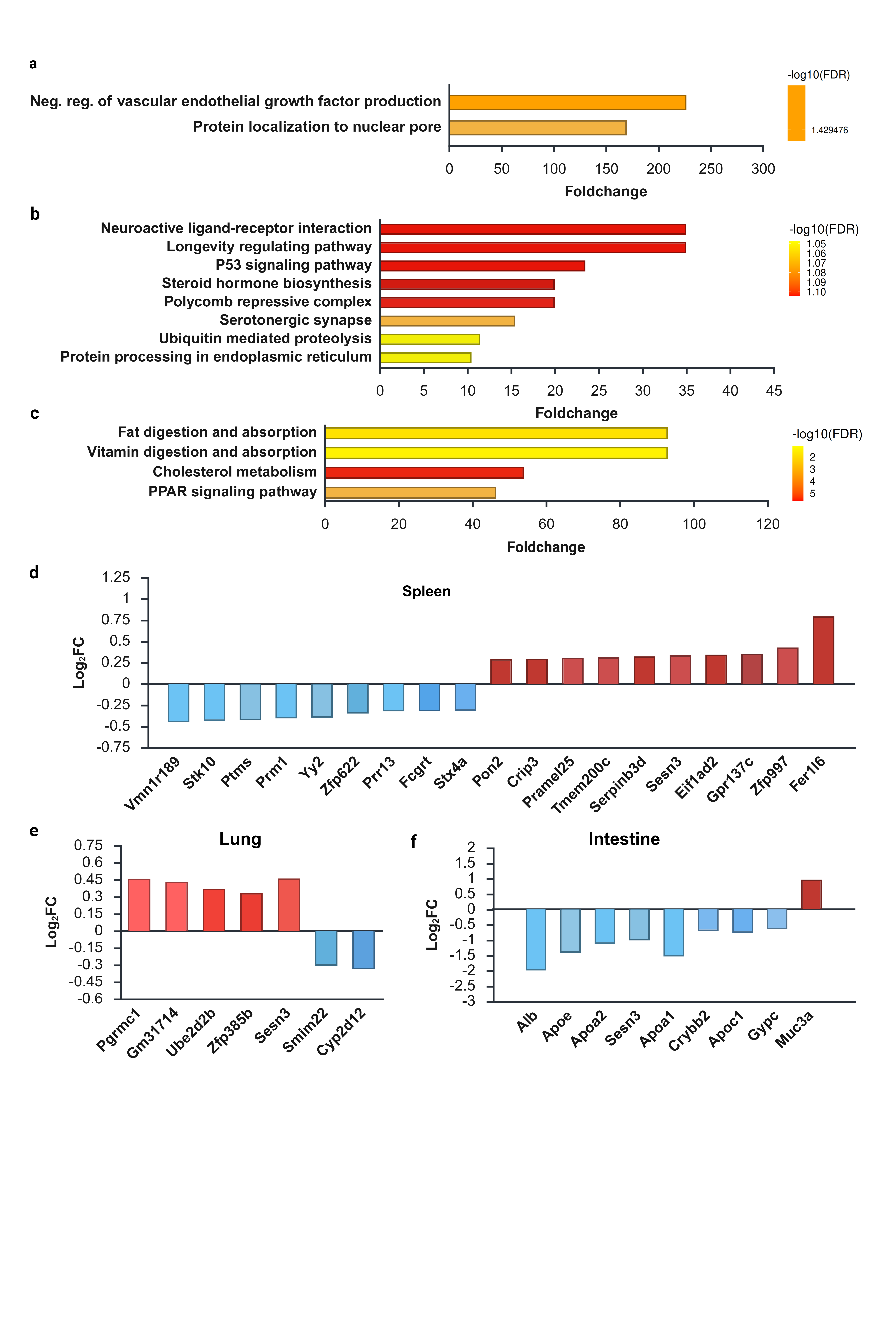


Supplementary Figure 2. Pathway analysis of DEGs of spleen, lung and intestine of mice treated with zoledronate.

Differentially expressed genes (DEGs) were identified via RNA-seq from the organs of aged mice following zoledronate (ZOL) treatment. **a–c** Bar plots illustrating Gene Ontology (GO) biological process enrichment for DEGs in the (**a**) spleen, (**b**) lung, and (**c**) intestine. **D** Visualization of the top 10 significantly up-regulated and down-regulated DEGs in the spleen of ZOL-treated mice versus age-matched controls. **e, f** Comprehensive profiling of all identified DEGs in the (**e**) lung and (**f**) intestine following ZOL administration.

Supplementary Figure 3. Uptake efficiency of fluorescent bisphosphonates in non-skeletal cells.

Non-skeletal cells, including AC-16, HEK, A549, PNT2, BMSCs, HCMVEC and HUH-7 cells, were treated with 0.1μΜ FAM-ZOL for 48 hours. After imaging, the uptake efficiency was quantified by the average fluorescent intensity of FAM-ZOL from the visual field in each cell group, which was calculated and illustrated as a ratio to that of A549 cells (n=6).


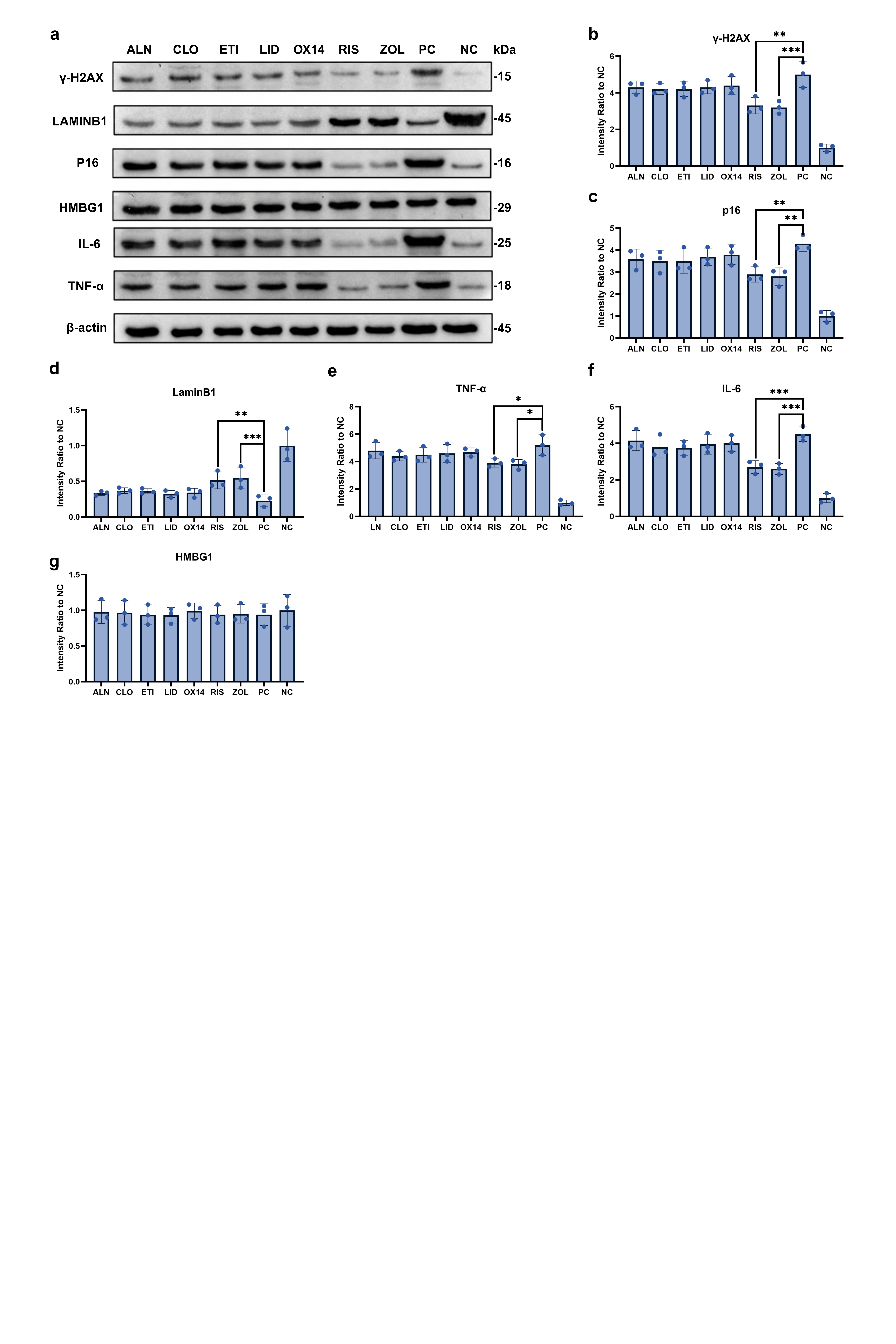


Supplementary Figure 4. Quantitation of senescence markers.

**a** Western blot image of senescence markers. **b-g** Quantitation of relative expression level of senescence markers (γ-H2AX (**b**), P16 (**c**), LaminB1(**d**), TNF-α (**e**), IL-6(**f**), HMBG1(**g**)) following BP-pretreatment and MMC induction (AC-16 cells, PC: 0.05μg/ml MMC; NC: vehicle; β-actin: internal control. * p<0.05, **p<0.01, ***p<0.001 vs positive control (PC). Data presented as mean±SD, n=3.


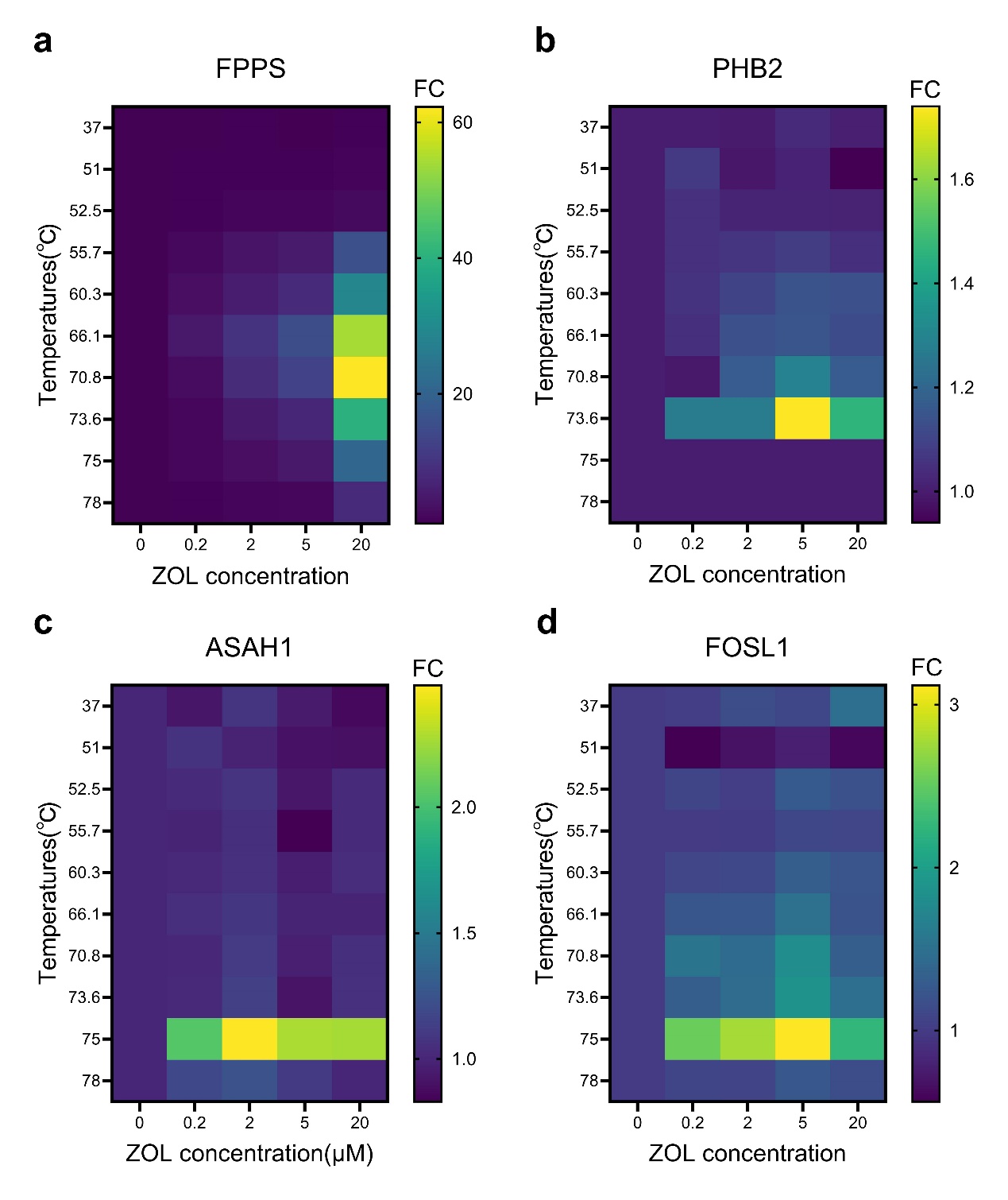


Supplementary Figure 5. Intensity heatmap of top ZOL stabilized targets following 2D thermal profiling.

Heat maps showing relative fold changes (FC) of protein abundance for FPPS(**a**), PHB2(**b**), ASAH1(**c**) and FOSL1(**d**) upon treatment with ZOL (0.2, 2, 5 and 20 μM) compared to DMSO treated lysate (first column on each plot) with increasing temperature (*y*-axis: 37, 51, 52.5, 55.7, 60.3, 66.1, 70.8, 73.6, 75, and 78 °C), FC: fold change.

Supplementary Figure 6. MEF2A target profile in mice heart RNAseq and human proteomics data.

MEF2A target genes were compiled from experimentally validated transcription factor–target interaction databases (ChEA, Cistrome, ENCODE, and TTRUST) and their expression patterns were examined in mouse heart RNA-seq data (zoledronate treatment versus control) and human plasma proteomics data (before and after 18 months of zoledronate infusion). Only differentially expressed genes or proteins with adjusted *p* < 0.05 were included following target mapping. Differential expression profiles are presented as bar plots of log2 fold change (log2FC).

**Supplementary Tables**

Supplementary Table 1. List of randomized clinical trials reporting mortality reductive effects of bisphosphonates.

| **Reference**  (author, date) | **Death (n/N)** | | **BPs** | **Sex (male/female), n** | **Risk Ratio**  **(95%CI)** |
| --- | --- | --- | --- | --- | --- |
|  | **Treatment** | **Placebo Control** |  |  |  |
| Lyles et al^4^ 2007 | 101/1054 | 141/1057 | Zoledronate | F:1619; M:508 | 0.72(0.56-0.91) |
| Reid et al^5^ 2018 | 27/1000 | 41/1000 | Zoledronate | F:2000 | 0.66(0.41-1.06) |
| Harris et al^6^ 1999 | 15/813 | 16/815 | Risedronate | F:1628 | 0.94(0.47-1.89) |
| Reginster et al^7^ 2000 | 11/407 | 17/407 | Risedronate | F:814 | 0.65(0.36-1.36) |
| McClung et al^8^ 2001 | 167/3104 | 178/3134 | Risedronate | F:6238 | 0.95(0.77-1.16) |
| Cummings et al^9^ 1998 | 37/2214 | 40/2218 | Alendronate | F:4432 | 0.93(0.59-1.44) |
| Ravn et al 1996 | 1/150 | 1/30 | Ibandronate | F:180 | 0.20(0.01-3.11) |
| Chesnut et al^11^ 2004 | 19/1954 | 10/975 | Ibandronate | F:2929 | 0.95(0.44-1.73) |
| Recker et al^12^ 2004 | 23/1911 | 13/949 | Ibandronate | F:2860 | 0.88(0.45-1.73) |
| Smerud et al^13^ 2012 | 0/66 | 3/63 | Ibandronate | F:129 | 0.14(0.01-2.59) |
| Boonen et al^14^ 2012 | 15/588 | 18/611 | Zoledronate | F:1199 | 0.87(0.44-1.70) |
| Nakamura et al^15^ 2017 | 2/333 | 3/332 | Zoledronate | F:621; M:40 | 0.66(0.41-1.06) |

This table provides a comprehensive summary of published randomized clinical trials that have reported on the association between bisphosphonate treatment and mortality reduction. For each trial, the table includes the first author, year of publication, number of patients (death, total) in the treatment and placebo control arms, the specific bisphosphonate used, the number of male and female participants and risk ratio.

Supplementary Table 2. List of pre-clinical and clinical studies showing the novel benefits of bisphosphonates on multiple non-osteoporotic diseases.

| **Author** | **Evidence** | **Disease** | **Bisphosphonates** | **Effects** |
| --- | --- | --- | --- | --- |
| Zameer et al^16^  Zameer et al^17^ | Pre-clinical  (mice, both sex) | Alzheimer’s disease  Huntington’s disease | ALN(1.76 mg/kg) and ZOL (0.5–1 mg/kg) | ⬆Cognitive and neurological function |
| Richard et al^18^ | Pre-clinical  (mice, both sex) | Hearing loss | ZOL(0.5 mg/kg) | ⬆Regeneration of cochlear synapses |
| Deborah et al ^19^  Tuomela et al^20^ | Pre-clinical  (human cell/mice) | Lymphoma | ZOL(100 µM) and ALN (0.5 mg/kg) | ⬆Apoptosis of malignant B cells. |
| Brent et al^21^  Fournieret al^22^  Vestergaard^23^ | Clinical  (female) | Breast cancer | CLO(800–1600 mg), PAM (90 mg), ZOL(4mg), IBAN (3 mg), ALN (70 mg), RIS (5 mg), ETI (400 mg) and TIL (400 mg) | ⬇Metastasis, risk and SRE |
| Sugie et al^24^ | Pre-clinical  (human/female) | Breast cancer | ZOL(4mg) | ⬆Expansion of γδ T cells |
| Mok et al^25^ | Clinical  (both sex) | Ankylosing spondylitis | PAM (60mg) | ⬇Pain and ⬆quality of life |
| Cubria et al^26^ | Pre-clinical  (mouse, both sex) | Hutchinson-Gilford  progeria | ZOL(200μg/kg) | ⬇Musculoskeletal phenotype of the disease |
| Gordon et al^27^ | Clinical  (both sex) | Hutchinson-Gilford  progeria | ZOL | ⬆Cardiovascular benefit and BMD |
| Tuesley^28^  Shigemitsu et al^29^  Zhang et al^30^ | Clinical  (female) | Endometrial/cervical  /ovarian cancer | ALN, ZOL (4mg) and RIS | ⬇Pain & metastasis |
| Lavoué et al^31^  Sawada^32^  Abdullah^33^ | Pre-clinical  (human cells) | Endometrial/cervical  /ovarian cancer | ZOL (100μM), ALN (100μM) and RIS | ⬇Migration & metastasis  ⬆Apoptosis |
| Coe et al^34^  Anastasilakis^35^ | Pre-clinical  (mice, both sex) | Diabetes | ALN (0.1 mg/kg) and ZOL | ⬇Risk and improve fasting plasma glucose, HbA1c, and insulin indices |
| Chen et al^36^  Jude et al^37^  Karimi et al^38^ | Clinical  (both sex) | Diabetes | ALN (70 mg), PAM (90 mg) and ZOL | ⬇Symptoms and disease activity |
| Modi et al^39^ | Pre-clinical  (human, both sex) | Multiple myeloma | CLO, ZOL(4mg), PAM (90 mg) and INC | ⬇Cancer cell growth, survival and microenvironment |
| Djulbegovic et al^40^ | Clinical  (both sex) | Multiple myeloma | CLO (1600mg) and PAM (90mg) | ⬇Risk, metastasis, SRE |
| Chiarella et al^41^ | Pre-clinical  (human cell) | Leukemia | ZOL(μM range) | ⬇Clonal expansion and maturation block of progenitor cells in myeloid hematological malignancies |
| Ganguly et al^42^ | Clinical  (both sex) | Leukemia | ZOL (4mg) | ⬇SRE |
| Janakiraman^43^  Lee et al^44^  Iolascon et al^45^  Barrera et al^46^ | Pre-clinical  (human, human cell and mice, both sex) | Rheumatoid arthritis | ALN (0.1 mg/kg), ZOL (10 µM), NER (25 mg IM/100mg IV) and CLO (5 mg/liposome) | ⬇Angiogenesis, inflammation and bone lesion |
| Valleala et al^47^  Muratore et al^48^  Xie et al^49^  Soejima et al^50^ | Clinical  (both sex) | Rheumatoid arthritis | ALN(5mg), ZOL(5mg), NER (25mg) and CLO (800mg) | ⬇Bone erosion, ⬇Atrophy, ⬇Inflammation |
| Lin et al^51^  Bo et al^52^ | Clinical  (both sex) | Stroke | ALN and PAM | ⬇Risk and pain |
| Banai et al^53^ | Clinical  (both sex) | Restenosis | ALN (1 mg/kg, liposome) | ⬆Treatment effects in patients with intense inflammation |
| Gutman et al^54^ | Pre-clinical  (Male rat) | Restenosis | ALN (1 mg/kg, liposome) | ⬆Anti-inflammatory effects |
| Reid et al^55^  Chen et al^56^  Teronen^57^ | Pre-clinical  (male rat and cell) | Vascular calcification/  atherosclerosis/  myocardial infarction | ALN(1 mg/kg), ETI, RIS, ZOL, IBAN, PAM and NER (10-100μM) | ⬆Endothelial function  ⬇MMP |
| Santos et al^58^  Kranenburg^59^  Caffarelli^60^ | Clinical  (both sex) | Vascular calcification/ atherosclerosis/  myocardial infarction | ALN, ETI, RIS, ZOL, IBAN, PAM and NER | ⬇Risk and symptoms |
| Kuriyama et al^61^ | Clinical  (male) | Gorham-Stout disease | ZOL (0.05 mg/kg I), CLO, ETI and PAM | ⬇Osteolysis, angiomatosis, and soft tissue swelling |
| Brufsky et al^62^ | Pre-clinical  (human, both sex) | SARS-CoV-2 | ZOL | Immunostimulant and DC modulator |
| Jeffrey et al^63^ | Clinical  (human, both sex) | SARS-CoV-2 | ALN and ZOL | ⬇testing, diagnosis and hospitalization |
| Mbese et al^64^ | Pre-clinical | Lung cancer | PAM and ZOL | ⬇Migration ⬇Metastasis  ⬆Apoptosis |
| Nakajima et al^65^  Mahtani et al^66^  Lopez-Olivo^67^  Li et al^68^ | Clinical  (both sex) | Lung cancer | ALN, CLO, IBAN, TIL, PAM (90mg) and ZOL(4mg) | ⬆Survival  ⬇Risk and metastasis |
| Sanz et al^69^ | Pre-clinical  (human cell) | HIV | ZOL(5μM) | Reactivate the latent reservoir |
| Prochaska et al^70^ | Clinical | Kidney Stone | ALN and RIS | ⬇Risk |
| Lang et al^71^ | Pre-clinical  (human, both sex) | Renal cell carcinoma | ZOL(4 mg) | ⬆Immune reaction |
| Broom et al^72^ | Clinical  (both sex) | Renal cell carcinoma | ZOL(4mg) | ⬇Risk and metastasis |
| Ang et al^73^ | Pre-clinical  (human cell) | Digestive cancer | ALN, ZOL, PAM, ETI, IBAN and RIS (10-100μM) | ⬇Migration ⬇Metastasis  ⬆Apoptosis |
| Ang et al^73^ | Clinical  (both sex) | Digestive cancer | ALN, ZOL, PAM, ETI, IBAN and RIS | ⬇Risk  Potential adjuvant |
| Saad et al^74^  Mbese et al^64^  Massey et al^75^  Iguchi et al^76^  Dumon et al^77^ | Pre-clinical  (human cell and male mice) | Prostate cancer | ZOL, ALN, PAM CLO and RIS (10-100μM) | ⬇Migration ⬇Metastasis  ⬆Apoptosis |
| Liu et al^78^ | Clinical  (both sex) | Prostate cancer | ZOL, ALN, PAM, CLO and RIS | ⬆Survival  ⬇Risk and metastasis |
| Duque et al^79^ | Clinical  (female) | Bone marrow adiposity | RIS (35 mg) | ⬇Bone marrow fat |
| Li^80^ | Pre-clinical  (female rat) | Bone marrow adiposity | ZOL (100 μg/kg) | Reverse marrow adipogenesis occurring during estrogen deficiency |
| Van et al^81^ | Clinical and Pre-clinical  (human, cell, mice, both sex) | Giant cell tumor | ZOL(4mg, 1-100μM, 0.05–0.2 mg/kg) | Controlling disease progression |
| Lu et al^82^ | Pre-clinical  (human cell, mice and rat, both sex) | Osteosarcoma | ZOL(1-100μM, 0.05–0.2 mg/kg) | ⬇Migration ⬇Metastasis  ⬆Apoptosis |
| Branco et al^83^ | Pre-clinical  (parasitic protozoa and helminths) | Parasite disease | RIS, IBAN and PAM (1-100μM) | ⬇Growth and viability of these parasites |
| Iris et al^84^ | Clinical  (both sex) | Pseudoxanthoma elasticum | ETI (400mg) | ⬇Arterial Calcification |

Both clinical and pre-clinical studies describing novel effects of bisphosphonates are summarized. The columns present the information of the first author, evidence type (clinical or pre-clinical with gender and model tested), disease, bisphosphonate (available dosage info) and effects shown in a specific study. ALN: alendronate; CLO: clodronate; ETI: etidronate, RIS: risedronate, ZOL: zoledronate, PAM: pamidronate, IBAN: ibandronate, TIL: tiludronate, NER: neridronate.

Supplementary Table 3. HAGR mapped results in female osteopenic patients treated with Zoledronate

| **Gene Symbol** | **Database** | **Gene Symbol** | **Database** |
| --- | --- | --- | --- |
| **SHC1** | GenAge | **FOXO3** | LongevityMap |
| **STAT3** | GenAge | **G6PD** | LongevityMap |
| **PLCG2** | GenAge | **GPX1** | LongevityMap |
| **PTEN** | GenAge | **ITGA1** | LongevityMap |
| **HSP90AA1** | GenAge | **JAK2** | LongevityMap |
| **NFKB1** | GenAge | **LYN** | LongevityMap |
| **PRKCA** | GenAge | **NFKB1** | LongevityMap |
| **FOXO3** | GenAge | **PDPK1** | LongevityMap |
| **FOXM1** | GenAge | **PIK3CA** | LongevityMap |
| **GPX1** | GenAge | **PIK3R1** | LongevityMap |
| **HSPD1** | GenAge | **PRKCA** | LongevityMap |
| **PTK2B** | GenAge | **PRKCB** | LongevityMap |
| **MAPK14** | GenAge | **RAF1** | LongevityMap |
| **PIK3R1** | GenAge | **SHC1** | LongevityMap |
| **JAK2** | GenAge | **SYK** | LongevityMap |
| **MAX** | GenAge | **TFAM** | LongevityMap |
| **PDPK1** | GenAge | **LONP1** | LongevityMap |
| **STUB1** | GenAge | **STX8** | LongevityMap |
| **CDC42** | GenAge | **PRDX3** | LongevityMap |
| **PIK3CA** | GenAge | **PNKP** | LongevityMap |
| **NFE2L1** | GenAge | **GHRL** | LongevityMap |
| **EHD4** | GenAge | **ALDOA** | CellAge |
| **MSN** | GenAge | **ARRB1** | CellAge |
| **RAB32** | GenAge | **ATG5** | CellAge |
| **PTPN6** | GenAge | **DUSP3** | CellAge |
| **SRGN** | GenAge | **EIF4G2** | CellAge |
| **EZR** | GenAge | **FASN** | CellAge |
| **PKP2** | GenAge | **FOXM1** | CellAge |
| **RAB31** | GenAge | **FOXO3** | CellAge |
| **PDLIM1** | GenAge | **G6PD** | CellAge |
| **STAT6** | GenAge | **GAPDH** | CellAge |
| **RRBP1** | GenAge | **HSP90AA1** | CellAge |
| **DUSP3** | GenAge | **ILK** | CellAge |
| **STAT1** | GenAge | **IRAK4** | CellAge |
| **DERL1** | GenAge | **JAK2** | CellAge |
| **ALDH6A1** | GenAge | **MAPK14** | CellAge |
| **PDHX** | GenAge | **MSN** | CellAge |
| **UBL4A** | GenAge | **MTHFD2** | CellAge |
| **PGK1** | GenAge | **PAK4** | CellAge |
| **COX5A** | GenAge | **PDPK1** | CellAge |
| **GPX7** | GenAge | **PIK3CA** | CellAge |
| **HOMER1** | GenAge | **PRKAA2** | CellAge |
| **PRDX3** | GenAge | **PTEN** | CellAge |
| **PAWR** | GenAge | **PTPN6** | CellAge |
| **VPS25** | GenAge | **RAF1** | CellAge |
| **PEX14** | GenAge | **SMAD1** | CellAge |
| **NAA10** | GenAge | **SMAD2** | CellAge |
| **ATG5** | GenAge | **SMAD3** | CellAge |
| **ILK** | GenAge | **SPHK1** | CellAge |
| **AHCYL1** | GenAge | **SRC** | CellAge |
| **G6PD** | GenAge | **STAT1** | CellAge |
| **AKT2** | GenAge | **STAT3** | CellAge |
| **ATG5** | GenDR | **STAT6** | CellAge |
| **FOXO3** | GenDR | **STIM1** | CellAge |
| **SERPINH1** | GenDR | **STUB1** | CellAge |
| **DECR1** | GenDR | **SYK** | CellAge |
| **ABLIM3** | GenDR | **WWP1** | CellAge |
| **CBL** | LongevityMap | **YWHAB** | CellAge |
| **FHIT** | LongevityMap | |  |

This table shows the mapped protein related genes in HAGR database and their corresponding sub-database name. GenAge is the benchmark database of genes related to ageing; The LongevityMap serves as a repository of genetic association studies of longevity and reflects our current knowledge of the genetics of human longevity. GenDR is a curated database of genes associated with dietary restriction in model organisms either from genetic manipulation experiments or gene expression profiling.CellAge is a database of genes associated with cell senescence.

Supplementary Table 4. ROI criteria selection in murine organs

This table shows the organ and corresponding tissue type extracted within the organ for the region of interest for spatial transcriptomic analysis with Nanostring GeoMx DSP platform.

| **Organ** | **Region within Organ** |
| --- | --- |
| Heart | Heart muscle tissue |
| Small Intestine | Differentiated epithelial |
|  | Crypts |
|  | Stroma |
|  | Smooth muscle |
| Liver | Central vein region |
|  | Periportal region |
| Pancreas | Ducts |
|  | Acinar |
|  | Islets |
| Kidney | Glomerulus |
|  | Collecting duct  proximal tubules |
|  | Distal tubules  Loop of Henle |
| Lungs | Bronchi |
|  | Alveoli |
|  | Stroma |
| Spleen | Peri-arteriolar lymphoid sheet |
|  | Mantle zone |
|  | Red pulp |

Supplementary Table 5. DEGs of liver in ZOL-treated ageing mice.

| **Gene** | **log_2_FC** | **padj** | **Gene** | **log_2_FC** | **padj** | **Gene** | **log_2_FC** | **padj** |
| --- | --- | --- | --- | --- | --- | --- | --- | --- |
| Acox1 | 1.42 | 2.32E-09 | Mmut | 0.55 | 0.0058 | Esrp2 | 0.52 | 0.0185 |
| Inhbe | 1.21 | 1.25E-08 | Tmem120a | 0.77 | 0.0058 | Dbp | 0.76 | 0.02287 |
| Erdr1 | 0.96 | 2.11E-06 | Cyp4a14 | 1.53 | 0.00583 | Nudt7 | 0.53 | 0.02612 |
| Slc38a4 | 0.99 | 9.04E-06 | Acadm | 0.77 | 0.00589 | Hadh | 0.60 | 0.02612 |
| Scd1 | 2.47 | 1.26E-05 | Pik3r1 | 0.68 | 0.00624 | Nr1h4 | 0.66 | 0.02771 |
| Nr1i3 | 0.92 | 2.81E-05 | Tns1 | 0.68 | 0.00624 | Acaa1a | 0.49 | 0.02771 |
| Acat1 | 0.76 | 3.47E-05 | LOC118568705 | 0.55 | 0.00631 | Ces1g | 0.64 | 0.02771 |
| Tdo2 | 1.01 | 4.14E-05 | Scp2 | 0.77 | 0.00785 | Gchfr | 0.60 | 0.02838 |
| G0s2 | 1.02 | 0.00011 | Sidt2 | 0.57 | 0.00863 | Tcim | -0.67 | 0.02838 |
| Nr1d1 | 0.95 | 0.00011 | Inmt | 0.77 | 0.00886 | Phyh | 0.42 | 0.02838 |
| Car3 | 1.71 | 0.00011 | Sardh | 0.82 | 0.00909 | Eef2 | 0.65 | 0.02838 |
| Arrdc3 | 0.89 | 0.00011 | Zfp36l2 | 0.63 | 0.00936 | Rcl1 | 0.54 | 0.02838 |
| Cyp2b13 | 1.54 | 0.0002 | Phb2 | 0.61 | 0.00955 | Rpl18a | 0.58 | 0.02838 |
| Cd1d1 | 0.93 | 0.00037 | Sult2a5 | 0.68 | 0.00964 | Acot3 | 0.70 | 0.02838 |
| Chd3 | 0.69 | 0.0004 | Cers2 | 0.55 | 0.00981 | Rpl27a | 0.52 | 0.03048 |
| Sgk1 | -1.17 | 0.0004 | Serpina3k | 1.28 | 0.01062 | Sphk2 | 0.53 | 0.03219 |
| Ttr | 0.82 | 0.00045 | Rps8 | 0.57 | 0.01156 | Rps6 | 0.41 | 0.03219 |
| Cyp4a10 | 1.39 | 0.00045 | Slc17a2 | 0.59 | 0.01216 | Ubald2 | 0.53 | 0.03219 |
| Ech1 | 0.66 | 0.00077 | Rpl36a | 0.56 | 0.01342 | Mgst1 | 0.58 | 0.03219 |
| Gk | 0.80 | 0.00084 | Gm6570 | 0.67 | 0.01457 | Chaf1a | -0.94 | 0.03219 |
| Prox1 | 0.62 | 0.00084 | Retsat | 1.05 | 0.0156 | Aph1a | 0.60 | 0.03389 |
| Serpine1 | 2.08 | 0.00085 | Decr1 | 0.52 | 0.01585 | Gpt2 | 0.95 | 0.03587 |
| Pctp | 0.94 | 0.00085 | Cyb5a | 0.67 | 0.01789 | Pgm1 | 0.45 | 0.03617 |
| Nfia | 0.60 | 0.00101 | Rps3a1 | 0.48 | 0.01789 | Naca | 0.42 | 0.03645 |
| Slc27a2 | 0.63 | 0.00108 | Hsd17b11 | 0.74 | 0.01789 | Mrap | 0.62 | 0.03676 |
| Eif1 | 0.45 | 0.00108 | Sephs2 | 0.53 | 0.01789 | Fau | 0.64 | 0.03714 |
| Optn | 0.58 | 0.00112 | Pcx | 0.75 | 0.01789 | Usf2 | 0.58 | 0.04014 |
| Cyp2d26 | 0.75 | 0.00126 | Gpam | 0.64 | 0.01789 | Cox8a | 0.45 | 0.043 |
| Acaa2 | 0.60 | 0.00126 | Ehhadh | 1.02 | 0.01789 | Cox6c | 0.45 | 0.043 |
| Ly6e | -0.89 | 0.00131 | Hspa9 | 0.55 | 0.01789 | Gpld1 | 0.56 | 0.04439 |
| Ppara | 0.63 | 0.00134 | Tmbim6 | 0.54 | 0.01833 | Kng2 | 0.77 | 0.04653 |
| Cpt1a | 0.70 | 0.00232 | Zfp36l1 | 0.44 | 0.0185 | Mcrip2 | 0.51 | 0.04738 |
| Pex16 | 0.71 | 0.00278 | Ak2 | 0.61 | 0.0185 | Lsr | 0.58 | 0.04981 |
| Sod1 | 0.60 | 0.00399 | Cluh | 0.43 | 0.0185 | Blvrb | 0.58 | 0.04981 |
| Hacd3 | 0.66 | 0.00462 | Rplp1 | 0.71 | 0.0185 | Ncl | 0.48 | 0.04995 |

This table shows the significant DEGs of liver in aging mice receiving treatment with zoledronate compared to vehicle control. Columns include genes, the log₂ (Fold Change), and the Benjamini-Hochberg adjusted p-value for each comparison.

Supplementary Table 6. DEGs of heart in ZOL-treated ageing mice.

| **Gene** | **log_2_FC** | **padj** | **Gene** | **log_2_FC** | **padj** |
| --- | --- | --- | --- | --- | --- |
| **Cox6a2** | 0.61 | 4.84E-07 | **Hadhb** | 0.47 | 0.019276 |
| **Pln** | 0.59 | 0.00015 | **Slc25a3** | 0.41 | 0.019855 |
| **Fth1** | 0.59 | 0.00025 | **Pnpla2** | 0.58 | 0.020453 |
| **Fabp3** | 0.54 | 0.00074 | **Atp5b** | 0.34 | 0.020453 |
| **Ankrd1** | -1.72 | 0.00074 | **Ablim1** | 0.61 | 0.021326 |
| **Ech1** | 0.69 | 0.00124 | **Map1lc3a** | 0.55 | 0.021326 |
| **Corin** | 0.77 | 0.00411 | **Mdh1** | 0.36 | 0.023515 |
| **Rps2** | 0.52 | 0.00501 | **LOC118568792** | 0.44 | 0.030609 |
| **Sesn3** | 0.64 | 0.0055 | **Rpl31** | 0.40 | 0.031365 |
| **Gm52520** | 0.58 | 0.0055 | **LOC118568634** | 0.24 | 0.03195 |
| **Acaa2** | 0.60 | 0.00669 | **Sfi1** | 0.45 | 0.032008 |
| **Myh6** | 0.29 | 0.00977 | **Hadh** | 0.46 | 0.032008 |
| **Acadm** | 0.49 | 0.00977 | **Ndufs6** | 0.36 | 0.040145 |
| **Slc25a4** | 0.35 | 0.00977 | **Rps3** | 0.50 | 0.040145 |
| **Atp5g2** | 0.55 | 0.00977 | **Vmn2r46** | 0.56 | 0.040145 |
| **Rpl28** | 0.52 | 0.01132 | **Smc3** | 0.38 | 0.040145 |
| **Rgs5** | -0.70 | 0.01132 | **Eif1** | 0.32 | 0.040881 |
| **Ndufb10** | 0.60 | 0.01132 | **Rpl21** | 0.33 | 0.043175 |
| **Tacc2** | 0.60 | 0.01169 | **Rrad** | 0.55 | 0.04337 |
| **Rpl17** | 0.39 | 0.01169 | **Acat1** | 0.45 | 0.04379 |
| **Cox5b** | 0.36 | 0.01317 | **Ankrd23** | -0.83 | 0.044427 |
| **Mdh2** | 0.41 | 0.01317 | **Cox6c** | 0.36 | 0.045973 |
| **Pygb** | 0.46 | 0.01636 | **Cox6b1** | 0.42 | 0.045973 |
| **Atp5a1** | 0.34 | 0.01658 | **Cyp2d12** | -0.49 | 0.045973 |
| **Hadha** | 0.46 | 0.01663 | **Echs1** | 0.50 | 0.045973 |
| **Rps3a1** | 0.50 | 0.01806 | **Slc25a20** | 0.61 | 0.046122 |
| **Atp2a2** | 0.36 | 0.01928 | **Ldhb** | 0.33 | 0.046944 |

This table shows the significant DEGs of heart in aging mice receiving treatment with zoledronate compared to vehicle control. Columns include genes, the log₂ (Fold Change), and the Benjamini-Hochberg adjusted p-value for each comparison.

Supplementary Table 7. List of stabilized and destabilized proteins following ZOL treatment (2D thermal profiling)

| **Gene** | **Combined Score** | **Gene** | **Combined Score** |
| --- | --- | --- | --- |
| FPPS | 0.98 | WDR11 | -0.56 |
| PHB2 | 0.52 | TSPAN6 | -0.54 |
| FOSL1 | 0.48 | CDH13 | -0.54 |
| ASAH1 | 0.48 | HYPK | -0.52 |
| CLTB | 0.46 | SYPL1 | -0.46 |
| NPC2 | 0.46 | ACTN1 | -0.44 |
| PCYOX1 | 0.44 | HNRNPA2B1 | -0.44 |
| ARL15 | 0.44 | MTDH | -0.44 |
| HNRNPD | 0.44 | DENR | -0.42 |
| TMED4 | 0.42 | PDLIM4 | -0.42 |
| ENO1 | 0.42 | BID | -0.42 |
| PODXL | 0.4 | CSNK2B | -0.42 |
| H2AC4 | 0.38 | HMGA1 | -0.42 |
| SEPTIN7 | 0.38 | VAMP2 | -0.42 |
| BASP1 | 0.36 | SLC9A6 | -0.4 |
| RAB6D | 0.36 | EIF1B | -0.4 |
| SV2A | 0.34 | SH3BGRL | -0.4 |
| DNAJC5 | 0.34 | TGFBI | -0.4 |
| EIF3C | 0.34 | LGALS1 | -0.4 |
| SLC25A1 | 0.34 | LAMTOR1 | -0.4 |
| TSFM | 0.34 | RWDD1 | -0.4 |
| FMR1 | 0.34 | UBE2N | -0.4 |
| STMN1 | 0.34 | TVP23C | -0.4 |
| DPYSL3 | 0.32 | UBL4A | -0.4 |
| PCYOX1L | 0.32 | ARF1 | -0.38 |
| ERAP1 | 0.32 | YWHAB | -0.38 |
| LGALS8 | 0.32 | RNF181 | -0.38 |
| ZMPSTE24 | 0.32 | CD82 | -0.38 |
| TMBIM1 | 0.32 | HMGA2 | -0.38 |
| RPS6 | 0.32 | HIBADH | -0.36 |
| TOMM70 | 0.32 | H2AX | -0.36 |

The table lists the top 62 proteins with the most significant changes in thermal stability, as determined by 2D thermal proteome profiling of AC16 cells treated with zoledronate. Proteins are ranked by a combined score reflecting their binding interaction with zoledronate. A positive score indicates protein stabilization, while a negative score indicates destabilization.

Data.S1 Differentially expressed plasma proteins at 18 and 36 months post-treatment of Zoledronate.

This table contains the results of the differential expression analysis comparing plasma protein abundance at 18 months and 36 months relative to baseline (0 months). Columns include protein identifiers (Target Name, UniProt, EntrezGeneID, EntrezGeneSymbol), the log₂ (Fold Change), and the Benjamini-Hochberg adjusted p-value for each comparison. The table was uploaded as additional excel file.

Data.S2 Differential gene expression analysis results of AC16 cells treated with zoledronate versus control.

This table contains the results of differential gene expression analysis comparing gene expression of AC16 cells treated with low dosage of zoledronate for 4 days with vehicle control. Significant up regulated and down regulated genes (padj <0.05) were recorded in separate sub sheets. Columns include ENSEMBL, gene symbol, full name and log2fold change value. The table was uploaded as additional excel file.

Data.S3 Differential ATAC-seq peak analysis of AC16 cells treated with zoledronate versus control.

This table contains the results of differential ATAC-seq peak analysis comparing ATAC-seq peak related genes of AC16 cells treated with low dosage of zoledronate for 4 days with vehicle control. Significant up regulated and down regulated ATAC-seq peak-related genes (padj <0.05) were recorded in separated sub sheets. Columns include ENSEMBL, gene symbol, full name and log2fold change value. The table was uploaded as additional excel file. The table was uploaded as additional excel file.

Reference

1. Valdés-Tresanco, M. S., Valdés-Tresanco, M. E., Valiente, P. A. & Moreno, E. AMDock: a versatile graphical tool for assisting molecular docking with Autodock Vina and Autodock4. *Biol. Direct* **15**, 12 (2020).

2. Morris, G. M. *et al.* AutoDock4 and AutoDockTools4: Automated docking with selective receptor flexibility. *J. Comput. Chem.* **30**, 2785–2791 (2009).

3. Feyertag, F. & Huber, K. V. M. TP-MAP - an Integrated Software Package for the Analysis of 1D and 2D Thermal Profiling Data. *bioRxiv* 2021.02.22.432361 (2021) doi:10.1101/2021.02.22.432361.

4. Lyles, K. W. *et al.* Zoledronic acid and clinical fractures and mortality after hip fracture. *N. Engl. J. Med.* **357**, 1799–1809 (2007).

5. Reid Ian R. *et al.* Fracture Prevention with Zoledronate in Older Women with Osteopenia. *N. Engl. J. Med.* **379**, 2407–2416 (2018).

6. Harris, S. T. *et al.* Effects of risedronate treatment on vertebral and nonvertebral fractures in women with postmenopausal osteoporosis: a randomized controlled trial. Vertebral Efficacy With Risedronate Therapy (VERT) Study Group. *JAMA* **282**, 1344–1352 (1999).

7. Reginster, J. *et al.* Randomized trial of the effects of risedronate on vertebral fractures in women with established postmenopausal osteoporosis. Vertebral Efficacy with Risedronate Therapy (VERT) Study Group. *Osteoporos. Int.* **11**, 83–91 (2000).

8. McClung, M. R. *et al.* Effect of risedronate on the risk of hip fracture in elderly women. Hip Intervention Program Study Group. *N. Engl. J. Med.* **344**, 333–340 (2001).

9. Cummings, S. R. *et al.* Effect of Alendronate on Risk of Fracture in Women With Low Bone Density but Without Vertebral FracturesResults From the Fracture Intervention Trial. *JAMA* **280**, 2077–2082 (1998).

10. Ravn, P., Clemmesen, B., Riis, B. J. & Christiansen, C. The effect on bone mass and bone markers of different doses of ibandronate: a new bisphosphonate for prevention and treatment of postmenopausal osteoporosis: a 1-year, randomized, double-blind, placebo-controlled dose-finding study. *Bone* **19**, 527–533 (1996).

11. Chesnut, C. H. 3rd *et al.* Effects of oral ibandronate administered daily or intermittently on fracture risk in postmenopausal osteoporosis. *J. Bone Miner. Res.* **19**, 1241–1249 (2004).

12. Recker, R. *et al.* Insufficiently dosed intravenous ibandronate injections are associated with suboptimal antifracture efficacy in postmenopausal osteoporosis. *Bone* **34**, 890–899 (2004).

13. Smerud, K. T. *et al.* A 1-year randomized, double-blind, placebo-controlled study of intravenous ibandronate on bone loss following renal transplantation. *Am. J. Transplant.* **12**, 3316–3325 (2012).

14. Boonen, S. *et al.* Fracture risk and zoledronic acid therapy in men with osteoporosis. *N. Engl. J. Med.* **367**, 1714–1723 (2012).

15. Nakamura, T. *et al.* Efficacy and safety of once-yearly zoledronic acid in Japanese patients with primary osteoporosis: two-year results from a randomized placebo-controlled double-blind study (ZOledroNate treatment in Efficacy to osteoporosis; ZONE study). *Osteoporos. Int.* **28**, 389–398 (2017).

16. Zameer, S. *et al.* Alendronate reduces the cognitive and neurological disturbances induced by combined doses of d-galactose and aluminum chloride in mice. *J. Appl. Toxicol.*  **41**, 1779–1793 (2021).

17. Zameer, S., Najmi, A. K., Vohora, D. & Akhtar, M. Bisphosphonates: Future perspective for neurological disorders. *Pharmacol. Rep.*  **70**, 900–907 (2018).

18. Seist, R. *et al.* Regeneration of Cochlear Synapses by Systemic Administration of a Bisphosphonate. *Front. Mol. Neurosci.* **13**, 87 (2020).

19. Vasconcellos, D. V. *et al.* Anti-Tumour Effect of Zoledronate in Cells from B Chronic Lymphoytic Leukemia and Low-Grade Lymphoma Patients. *Blood* **104**, 4830–4830 (2004).

20. Tuomela, J. M., Valta, M. P., Väänänen, K. & Härkönen, P. L. Alendronate decreases orthotopic PC-3 prostate tumor growth and metastasis to prostate-draining lymph nodes in nude mice. *BMC Cancer* **8**, 81 (2008).

21. O’Carrigan, B. *et al.* Bisphosphonates and other bone agents for breast cancer. *Cochrane Database Syst. Rev.* **10**, CD003474 (2017).

22. Fournier, A. *et al.* Use of Bisphosphonates and Risk of Breast Cancer in a French Cohort of Postmenopausal Women. *J. Clin. Oncol.* **35**, 3230–3239 (2017).

23. Vestergaard, P., Fischer, L., Mele, M., Mosekilde, L. & Christiansen, P. Use of bisphosphonates and risk of breast cancer. *Calcif. Tissue Int.* **88**, 255–262 (2011).

24. Sugie, T. *et al.* Zoledronic acid-induced expansion of γδ T cells from early-stage breast cancer patients: effect of IL-18 on helper NK cells. *Cancer Immunol. Immunother.*  **62**, 677–687 (2013).

25. Mok, C. C., Li, O. C., Chan, K. L., Ho, L. Y. & Hui, P. K. Effect of golimumab and pamidronate on clinical efficacy and MRI inflammation in axial spondyloarthritis: a 48-week open randomized trial. *Scand. J. Rheumatol.* **44**, 480–486 (2015).

26. Cubria, M. B. *et al.* Evaluation of musculoskeletal phenotype of the G608G progeria mouse model with lonafarnib, pravastatin, and zoledronic acid as treatment groups. *Proc. Natl. Acad. Sci. U. S. A.* **117**, 12029–12040 (2020).

27. Gordon, L. B. *et al.* Clinical Trial of the Protein Farnesylation Inhibitors Lonafarnib, Pravastatin, and Zoledronic Acid in Children With Hutchinson-Gilford Progeria Syndrome. *Circulation* **134**, 114–125 (2016).

28. Tuesley, K. M. *et al.* Nitrogen-based Bisphosphonate Use and Ovarian Cancer Risk in Women Aged 50 Years and Older. *J. Natl. Cancer Inst.* **114**, 878–884 (2022).

29. Shigemitsu, A., Furukawa, N., Koike, N. & Kobayashi, H. Endometrial cancer diagnosed by the presence of bone metastasis and treated with zoledronic Acid: a case report and review of the literature. *Case Rep. Oncol.* **3**, 471–476 (2010).

30. Zhang, X. *et al.* Risk reduction of endometrial and ovarian cancer after bisphosphonates use: A meta-analysis. *Gynecol. Oncol.* **150**, 509–514 (2018).

31. Lavoué, V. *et al.* Sensitization of ovarian carcinoma cells with zoledronate restores the cytotoxic capacity of Vγ9Vδ2 T cells impaired by the prostaglandin E2 immunosuppressive factor: Implications for immunotherapy. *Int. J. Cancer* **131**, E449–E462 (2012).

32. Sawada, K. *et al.* Alendronate inhibits lysophosphatidic acid-induced migration of human ovarian cancer cells by attenuating the activation of rho. *Cancer Res.* **62**, 6015–6020 (2002).

33. Abdullah, M. I., Abed, M. N. & Richardson, A. Inhibition of the mevalonate pathway augments the activity of pitavastatin against ovarian cancer cells. *Sci. Rep.* **7**, 8090 (2017).

34. Coe, L. M., Tekalur, S. A., Shu, Y., Baumann, M. J. & McCabe, L. R. Bisphosphonate treatment of type I diabetic mice prevents early bone loss but accentuates suppression of bone formation. *J. Cell. Physiol.* **230**, 1944–1953 (2015).

35. Anastasilakis, A. D. *et al.* The Impact of Antiosteoporotic Drugs on Glucose Metabolism and Fracture Risk in Diabetes: Good or Bad News? *J. Clin. Med.* **10**, (2021).

36. Chen, P.-W. *et al.* Association of bisphosphonates with diabetes risk and glycemic control: a meta-analysis. *Osteoporos. Int.* **34**, 387–397 (2023).

37. Jude, E. B. *et al.* Bisphosphonates in the treatment of Charcot neuroarthropathy: a double-blind randomised controlled trial. *Diabetologia* **44**, 2032–2037 (2001).

38. Karimi Fard, M. *et al.* Alendronate improves fasting plasma glucose and insulin sensitivity, and decreases insulin resistance in prediabetic osteopenic postmenopausal women: A randomized triple-blind clinical trial. *J. Diabetes Investig.* **10**, 731–737 (2019).

39. Modi, N. D. & Lentzsch, S. Bisphosphonates as antimyeloma drugs. *Leukemia* **26**, 589–594 (2012).

40. Djulbegovic, B. *et al.* Bisphosphonates in multiple myeloma. *Cochrane Database Syst. Rev.* CD003188 (2001).

41. Chiarella, E., Nisticò, C., Di Vito, A., Morrone, H. L. & Mesuraca, M. Targeting of Mevalonate-Isoprenoid Pathway in Acute Myeloid Leukemia Cells by Bisphosphonate Drugs. *Biomedicines* **10**, (2022).

42. Ganguly, S. *et al.* Prophylactic use of zoledronic acid to prevent early bone loss is safe and feasible in patients with acute myeloid leukemia undergoing allogeneic stem cell transplantation. *Clin. Transplant.* **26**, 447–453 (2012).

43. Janakiraman, V. *et al.* Rheumatoid arthritis treatment with zoledronic acid, a potentialinhibitorofGWAS-derived pharmacogenetics STAT3 and IL2 targets. *Gene* **866**, 147338 (2023).

44. Lee, H., Bhang, S. H., Lee, J. H., Kim, H. & Hahn, S. K. Tocilizumab–Alendronate Conjugate for Treatment of Rheumatoid Arthritis. *Bioconjug. Chem.* **28**, 1084–1092 (2017).

45. Iolascon, G. & Moretti, A. The Rationale for Using Neridronate in Musculoskeletal Disorders: From Metabolic Bone Diseases to Musculoskeletal Pain. *Int. J. Mol. Sci.* **23**, (2022).

46. Barrera, P. *et al.* Synovial macrophage depletion with clodronate-containing liposomes in rheumatoid arthritis. *Arthritis Rheum.* **43**, 1951–1959 (2000).

47. Valleala, H., Laasonen, L., Koivula, M.-K., Risteli, J. & Konttinen, Y. T. Effect of oral clodronate on structural damage and bone turnover in rheumatoid arthritis. *Clin. Exp. Rheumatol.* **30**, 114–117 (2012).

48. Muratore, M., Quarta, E. & Quarta, L. Intramuscular neridronate in patients with rheumatoid arthritis using corticosteroids: evaluation of treatment adherence in a randomized, open-label comparison with other bisphosphonates. *Acta Biomed.* **84**, 23–29 (2013).

49. Xie, J. *et al.* Zoledronic acid ameliorates the effects of secondary osteoporosis in rheumatoid arthritis patients. *J. Orthop. Surg.* **14**, 421 (2019).

50. Soejima, M. *et al.* Therapeutic effects of alendronate on bone erosion and atrophy in a patient with rheumatoid arthritis and hepatitis C virus infection. *J. Rheumatol.* **35**, 2284–2286 (2008).

51. Lin, S.-M. *et al.* Association between bisphosphonate use and stroke risk: a meta-analysis. *Osteoporos. Int.* **34**, 1625–1636 (2023).

52. Bo, Z. *et al.* Pharmacotherapies for Central Post-Stroke Pain: A Systematic Review and Network Meta-Analysis. *Oxid. Med. Cell. Longev.* **2022**, 3511385 (2022).

53. Banai, S. *et al.* Targeted anti-inflammatory systemic therapy for restenosis: the Biorest Liposomal Alendronate with Stenting sTudy (BLAST)-a double blind, randomized clinical trial. *Am. Heart J.* **165**, 234-240.e1 (2013).

54. Gutman, D. & Golomb, G. Liposomal alendronate for the treatment of restenosis. *J. Control. Release.* **161**, 619–627 (2012).

55. Billington, E. O. & Reid, I. R. Benefits of Bisphosphonate Therapy: Beyond the Skeleton. *Curr. Osteoporos. Rep.* **18**, 587–596 (2020).

56. Chen, G.-P. *et al.* Chronic inhibition of farnesyl pyrophosphate synthase improves endothelial function in spontaneously hypertensive rats. *Biochem. Pharmacol.* **80**, 1684–1689 (2010).

57. Teronen, O. *et al.* MMP inhibition and downregulation by bisphosphonates. *Ann. N. Y. Acad. Sci.* **878**, 453–465 (1999).

58. Santos, L. L., Cavalcanti, T. B. & Bandeira, F. A. Vascular effects of bisphosphonates-a systematic review. *Clin. Med. Insights Endocrinol. Diabetes* **5**, 47–54 (2012).

59. Kranenburg, G. *et al.* Bisphosphonates for cardiovascular risk reduction: A systematic review and meta-analysis. *Atherosclerosis* **252**, 106–115 (2016).

60. Caffarelli, C., Montagnani, A., Nuti, R. & Gonnelli, S. Bisphosphonates, atherosclerosis and vascular calcification: update and systematic review of clinical studies. *Clin. Interv. Aging* **12**, 1819–1828 (2017).

61. Kuriyama, D. K., McElligott, S. C., Glaser, D. W. & Thompson, K. S. Treatment of Gorham-Stout disease with zoledronic acid and interferon-α: a case report and literature review. *J. Pediatr. Hematol. Oncol.* **32**, 579–584 (2010).

62. Brufsky, A., Marti, J. L. G., Nasrazadani, A. & Lotze, M. T. Boning up: amino-bisphophonates as immunostimulants and endosomal disruptors of dendritic cell in SARS-CoV-2 infection. *J. Transl. Med.* **18**, 261 (2020).

63. Thompson, J. *et al.* Association between bisphosphonate use and COVID-19 related outcomes. *eLife* **12**, (2023).

64. Mbese, Z. & Aderibigbe, B. A. Bisphosphonate-Based Conjugates and Derivatives as Potential Therapeutic Agents in Osteoporosis, Bone Cancer and Metastatic Bone Cancer. *Int. J. Mol. Sci.* **22**, (2021).

65. Nakajima, J. *et al.* A phase I study of adoptive immunotherapy for recurrent non-small-cell lung cancer patients with autologous gammadelta T cells. *Eur. J. Cardio-Thorac. Surg.* **37**, 1191–1197 (2010).

66. Mahtani, R., Khan, R. & Jahanzeb, M. The potential application of zoledronic acid as anticancer therapy in patients with non-small-cell lung cancer. *Clin. Lung Cancer* **12**, 26–32 (2011).

67. Lopez-Olivo, M. A. *et al.* Bisphosphonates in the treatment of patients with lung cancer and metastatic bone disease: a systematic review and meta-analysis. *Support. Care Cancer.* **20**, 2985–2998 (2012).

68. Li, M., Zhong, M. & Guan, C. Bisphosphonates and risk of lung cancer: Protocol for a systematic review and meta-analysis. *Medicine (Baltimore)* **100**, e22839 (2021).

69. Sanz, M. *et al.* Aminobisphosphonates reactivate the latent reservoir in people living with HIV-1. *bioRxiv : the preprint server for biology* 2023.02.07.527421.

70. Prochaska, M. Bisphosphonates and management of kidney stones and bone disease. *Curr. Opin. Nephrol. Hypertens.* **30**, 184–189 (2021).

71. Lang, J. M. *et al.* Pilot trial of interleukin-2 and zoledronic acid to augment γδ T cells as treatment for patients with refractory renal cell carcinoma. *Cancer Immunol. Immunother.* **60**, 1447–1460 (2011).

72. Broom, R. J. *et al.* Everolimus and zoledronic acid in patients with renal cell carcinoma with bone metastases: a randomized first-line phase II trial. *Clin. Genitourin. Cancer* **13**, 50–58 (2015).

73. Ang, C., Doyle, E. & Branch, A. Bisphosphonates as potential adjuvants for patients with cancers of the digestive system. *World J. Gastroenterol.* **22**, 906–916 (2016).

74. Saad, F. & Mulders, P. Bisphosphonate anticancer activity in prostate cancer and other genitourinary cancers. *Anticancer Agents Med. Chem.* **12**, 129–136 (2012).

75. Massey, A. S. *et al.* Potentiating the Anticancer Properties of Bisphosphonates by Nanocomplexation with the Cationic Amphipathic Peptide, RALA. *Mol. Pharm.* **13**, 1217–1228 (2016).

76. Iguchi, K., Tatsuda, Y., Usui, S. & Hirano, K. Pamidronate inhibits antiapoptotic bcl-2 expression through inhibition of the mevalonate pathway in prostate cancer PC-3 cells. *Eur. J. Pharmacol.* **641**, 35–40 (2010).

77. Dumon, J.-C., Journé, F., Kheddoumi, N., Lagneaux, L. & Body, J.-J. Cytostatic and apoptotic effects of bisphosphonates on prostate cancer cells. *Eur. Urol.* **45**, 521–8; discussion 528-529 (2004).

78. Liu, J. *et al.* Bisphosphonates in the Treatment of Patients With Metastatic Breast, Lung, and Prostate Cancer: A Meta-Analysis. *Medicine (Baltimore)* **94**, e2014 (2015).

79. Duque, G., Li, W., Adams, M., Xu, S. & Phipps, R. Effects of risedronate on bone marrow adipocytes in postmenopausal women. *Osteoporos. Int. J.* **22**, 1547–1553 (2011).

80. Li, G.-W. *et al.* Influence of early zoledronic acid administration on bone marrow fat in ovariectomized rats. *Endocrinology* **155**, 4731–4738 (2014).

81. van der Heijden, L., Dijkstra, S., van de Sande, M. & Gelderblom, H. Current concepts in the treatment of giant cell tumour of bone. *Curr. Opin. Oncol.* **32**, 332–338 (2020).

82. Lu, K.-H., Lu, E. W.-H., Lin, C.-W., Yang, J.-S. & Yang, S.-F. New insights into molecular and cellular mechanisms of zoledronate in human osteosarcoma. *Pharmacol. Ther.* **214**, 107611 (2020).

83. Branco Santos, J. C. *et al.* Bisphosphonate-Based Molecules as Potential New Antiparasitic Drugs. *Mol.* **25**, (2020).

84. Harmsen, I. M. *et al.* Cyclical Etidronate Reduces the Progression of Arterial Calcifications in Patients with Pseudoxanthoma Elasticum: A 6-Year Prospective Observational Study. *J. Clin. Med.* **13**, (2024).
